# Optimal COVID-19 Vaccine Sharing Between Two Nations That Also Have Extensive Travel Exchanges

**DOI:** 10.3389/fpubh.2021.633144

**Published:** 2021-08-12

**Authors:** Chris Huntingford, Thomas Rawson, Michael B. Bonsall

**Affiliations:** ^1^UK Centre for Ecology and Hydrology, Wallingford, United Kingdom; ^2^Mathematical Ecology Research Group, Department of Zoology, University of Oxford, Oxford, United Kingdom

**Keywords:** medical ethics, infectious travellers, disease transmission, epidemiology, SARS-CoV-2, COVID-19, vaccine, SIRV model

## Abstract

Countries around the world have observed reduced infections from the SARS-CoV-2 virus, that causes COVID-19 illness, primarily due to non-pharmaceutical interventions (NPIs) such as lockdowns and social distancing measures designed to limit physical proximity between people. However, economies and societal interactions require restarting, and so lockdowns cannot continue indefinitely. Therefore, much hope is placed in using newly developed vaccines as a route back to normality, but this raises key questions about how they are shared. There are also emerging questions regarding travel. For instance, international business and trade necessitates at least some in-person exchanges, alongside restarting travel also for tourist purposes. By utilising a Susceptible-Infected-Recovered-Vaccinated (SIRV) mathematical model, we simulate the populations of two nations in parallel, where the first nation produces a vaccine and decides the extent to which it is shared with the second. Overlaying our mathematical structure is the virus-related effects of travel between the two nations. We find that even with extensive travel, nation one minimises its total number of deaths by simply retaining vaccines, aiming for full inoculation as fast as possible, suggesting that the risks posed by travel can be mitigated by rapidly vaccinating its own population. If instead we consider the total deaths i.e., sum of deaths of both nations, then such a policy of not sharing by nation one until full vaccination is highly sub-optimal. A policy of low initial sharing causes many more deaths in nation two than lives saved in nation one, raising important ethical issues. This imbalance in the health impact of vaccination provision must be considered as some countries begin to approach the point of extensive vaccination, while others lack the resources to do so.

## 1. Introduction

Through 2020, countries across the world have worked to diminish the impact of the SARS-CoV-2 virus and lower the related levels of COVID-19 illness (1). Initially, these control measures have included the implementation of non-pharmaceutical interventions (NPIs) to keep people apart, such as “social distancing” policies of limited socialising, or “lockdowns” whereby citizens are instructed to remain at home. Such measures have been found to be broadly successful (2). During the Northern Hemisphere Autumn period, there was a lifting of many aspects of lockdown across Europe, and societies were encouraged to reopen. Although some social distancing measures remained in place to lower transmission, in many instances the predicted possibility of additional waves of infection (3, 4) occurred. In addition, there is much confusion over whether it is safe to travel between nations, resulting in rapidly changing policies of country-specific travel restrictions because of concerns over importing infections. Yet in one of the first papers to consider this, Chinazzi et al. (5) find that restrictions on travel achieve only small reductions in infections without additional actions to limit transmission within countries. Travel remains essential in a world composed of tightly interwoven economies. Exchange visits remain important between nations that are trading partners, and hence restrictions are detrimental to business advancement. COVID-19, as expected, is proving especially harmful to businesses that support travel, whether for work or tourism purposes (6).

Toward the end of year 2020, multiple research centres performed advanced stage trials of potential COVID-19 vaccines [e.g., (7–9)]. In the United Kingdom for instance, approval has been given for the vaccines produced by Pfizer/BioNTech (10), AstraZeneca (11), and Moderna (12). However, vaccine availability also raises new questions. Should a country discover a safe vaccine, followed rapidly by mass production, a key question is how should it be distributed? A reasonable working assumption is that to reduce infections to levels that would promote herd immunity and fade-out of disease, a substantial fraction of inoculations will be given to citizens of the country that developed the vaccine. If during vaccine production and distribution, that country (nation “one”) also implements measures to constrain infections while waiting for everyone to be vaccinated, then people travelling from another country (nation “two”) may be a concern. Such concerns will be warranted if nation two places less emphasis on restricting the spread of COVID-19. Additionally, the infections of citizens of nation one are likely to increase as they visit nation two. Hence where extensive trade-related travel exchanges between nation one and nation two are critical, a fundamental question is whether it is prudent for nation one to share vaccines with nation two before nation one is fully vaccinated. A related question is whether more lives are saved overall (i.e., considering the combined effect on nation one and nation two) by the early sharing of vaccines. Here we use a mathematical representation of virus transmission, vaccine provision and sharing, and travel between two nations, to investigate these questions.

## 2. Governing Equations

### 2.1. Infections, Deaths, Vaccinations, and Inter-Nation Exchange

Our aim is to provide a set of equations that are as simple as possible, yet retain sufficient complexity that they can describe three main effects of: (1) infection increases starting from low case numbers (e.g., after the lifting of lockdown measures), (2) travel between two nations and any related transfer of infections, and (3) the effects of different options for vaccine distribution. In our conceptual modelling framework, we consider two nations, “one” and “two” indexed by “1” and “2,” respectively, and with populations *N*_1_ and *N*_2_ (people). Each nation has a virus transmission rate β (new infections per day caused by an infected individual in a completely susceptible population), and an infected case fatality rate α, which is a fraction of those currently infected. To account for travel between the two nations, variable *f*_2_ is the fraction of the population of nation two visiting nation one at any given time. Similarly, for opposite travel, *f*_1_ is the fraction of nation one visiting nation two. Variables *f*_1_ and *f*_2_ are considered invariant. The assumption is that the exchange of people between the two countries, characterised by *f*_1_ and *f*_2_, is continuous and so all people will be available in their own nation at some point to receive any vaccine. We list all model parameters in Table 1. The simulation framework has some similarities to modelling different communities within a single country and during lockdown, such as those who have essential roles and continued to work, vs. those isolating [e.g., (13)]. Here, we are concerned with continuous travel-based exchange between communities (i.e., two nations) and in parallel with vaccine introduction.

**Table 1:** Parameters and initial conditions used for our governing Equations (1)–(4) and (7)–(10), and in Figure 1.

**Parameter symbol**	**Parameter name**	**Value**	**Units**
β_1_	Daily transmission rate, nation one	0.25	Day^−1^
β_2_	Daily transmission rate, nation two	0.25	Day^−1^
*f*_1_	Fraction of nation one visiting nation two	0.2	
*f*_2_	Fraction of nation two visiting nation one	0.2	
γ	Recovery rate	0.2	Day^−1^
σ	Rate of waning immunity (zero implies continued full immunity)	0.0	Day^−1^
α	Infected case fatality rate	0.005	Day^−1^
*Q*	Total vaccines produced per day	1 × 10^6^	Vaccines day^−1^
*V*_1_(0)	Initial number of people vaccinated, nation one	0	People
*V*_2_(0)	Initial number of people vaccinated, nation two	0	People
*S*_1_(0)	Initial number of people susceptible, nation one	49 × 10^6^	People
*S*_2_(0)	Initial number of people susceptible, nation two	49 × 10^6^	People
*I*_1_(0)	Initial number of people infected, nation one	0.2 × 10^6^	People
*I*_2_(0)	Initial number of people infected, nation two	0.2 × 10^6^	People
*R*_1_(0)	Initial number of people recovered, nation one	0.8 × 10^6^	People
*R*_2_(0)	Initial number of people recovered, nation two	0.8 × 10^6^	People

*Perturbations to these parameters are that f_1_ = f_2_ = 0.05 is tested and presented as a sensitivity analysis to less travel in Figure 2. Then in Figures 3B,D, we set β_2_ = 0.5 as a sensitivity estimate of nation two having a far higher transmission rate. Further perturbations are made in Supplementary Figures 1–6*.

We utilise a form of bulk compartmental model to describe COVID-19 transmission. Such models have proven effective in modelling the spread of infectious disease for almost a century, since e.g., (14). Our first equation characterises the number of susceptibles, *S* (people) in nation one (*S*_1_). The rate of change of *S*_1_ in time *t* (specified as days since the start of vaccine production), is given by Equation (1), and has four terms on the right-hand side. The first describes the number of citizens of nation one who become infected while located in nation one, and this includes the impact of increased infection rates due to visitors from nation two (i.e., the *f*_2_*I*_2_ term). These people leave the susceptibility group and enter the infectious group. The second term is those from nation one, but visiting nation two, and who become infected while overseas. The third is the re-entry of those who have recovered from the illness, characterised by rate of waning immunity, σ (day^−1^), and where *R* (people) is the number who have recovered from COVID-19. A value of σ = 0 is valid if it is found that those who have recovered from the virus also have long-term complete immunity. We assume in our main calculations full immunity (i.e., σ = 0), although below in numerical results, we also perform a factorial simulation with a small value σ > 0, based on emerging literature. The last term is the impact of vaccination. Variable *Q* (vaccines day^−1^) is the total number of vaccinations produced each day, available for use in either nation one, nation two, or sharing between the two countries. Available vaccines are assumed to be used immediately, and distributed according to the fraction of susceptible people and those who have recovered, *R*. Hence, despite strong immunity, we additionally assume that out of caution, the recovered group is offered and accepts vaccines. Critically, for the analysis here, quantity ν_1_(*t*) is the fraction of vaccines retained for use by nation one, and that may vary in time. It is different time histories of this variable, ν_1_(*t*), that we test for their impact in our simulation framework. These equation terms combine, respectively, to give for nation one:

(1) $$\begin{array}{l} \frac{\text{d}S_{1}}{\text{d}t}=-\beta_{1}\frac{(1-f_{1})S_{1}}{(1-f_{1})N_{1}+f_{2}N_{2}}\left[(1-f_{1})I_{1}+f_{2}I_{2}\right]\\ \;-\beta_{2}\frac{f_{1}S_{1}}{f_{1}N_{1}+(1-f_{2})N_{2}}\left[(1-f_{2})I_{2}+f_{1}I_{1}\right]\\ \;+\sigma R_{1}-\nu_{1}Q\frac{S_{1}}{S_{1}+R_{1}}. \end{array}$$

As susceptible people are infected, they move to the infected group, *I* (people). People leave the infected group by recovery as described by a rate γ (day^−1^), or by dying and corresponding to a mortality rate α (day^−1^). Hence, for nation one, these changes to infections (*I*_1_) are given by:

(2) $$\begin{array}{l} \frac{\text{d}I_{1}}{\text{d}t}=\beta_{1}\frac{(1-f_{1})S_{1}}{(1-f_{1})N_{1}+f_{2}N_{2}}\left[(1-f_{1})I_{1}+f_{2}I_{2}\right]\\ \;+\beta_{2}\frac{f_{1}S_{1}}{f_{1}N_{1}+(1-f_{2})N_{2}}\left[(1-f_{2})I_{2}+f_{1}I_{1}\right]\\ \;-\gamma I_{1}-\alpha I_{1}. \end{array}$$

The inverse of γ is the period, in days, that a person is infectious. From the value presented in Table 1, this gives a period of 5 days (15). Others suggest longer infection periods of a median of 8 days (16), or a range of 7–14 days (17). Very early during the emergence of the COVID-19 illness, it was realised that approximately one third of infected people show no signs of illness (18) yet these people can still infect others (19). Such asymptomatic individuals are included in our *I*_1_ and *I*_2_ groups, and so these quantities are not simply people who are unwell.

The recovery group, *R*, increases in size based on those who were previously infected and survive. People return to the susceptible group if there is no lifelong immunity effects or that immunity is time-limited, as characterised by parameter σ. People also leave the recovered group if vaccinated. For nation one, the number of recovered individuals (*R*_1_) is:

(3) $$\frac{\text{d}R_{1}}{\text{d}t}=\gamma I_{1}-\sigma R_{1}-\nu_{1}Q\frac{R_{1}}{S_{1}+R_{1}}.$$

Again, it is the last term on the right-hand side of Equation (3) that captures the assumption noted above, that even when full immunity is assumed (i.e., σ = 0), a cautious approach is taken of vaccinating those who have recovered. Finally, the group of people vaccinated, *V*, for the first nation (*V*_1_) satisfies:

(4) $$\frac{\text{d}V_{1}}{\text{d}t}=\nu_{1}Q.$$

In the set of governing equations for nation one, we assume that all people are in one group: *S, I, R*, or *V*, and so:

(5) $$N_{1}=S_{1}+I_{1}+R_{1}+V_{1}.$$

In Equations (1)–(4), there is a final implicit assumption that births and non-COVID-19 deaths balance. Hence these equations, when combined additively, give d*N*_1_/d*t* = −α*I*_1_, which is the excess death rate due to COVID-19 illness. The total number to have died from COVID-19, *D* (people), in nation one and after time *t* is, therefore:

(6) $$D_{1}(t)=\int_{0}^{t}\alpha I_{1}(t^{\prime })\text{d}t^{\prime }.$$

For the second nation, then the governing equations are very similar to those of Equations (1)–(4); the indices are swapped, and the fraction of vaccines ν_1_ is now replaced by 1 − ν_1_. For completeness, we write these equations out below:

(7) $$\begin{array}{l} \frac{\text{d}S_{2}}{\text{d}t}=-\beta_{2}\frac{(1-f_{2})S_{2}}{(1-f_{2})N_{2}+f_{1}N_{1}}\left[(1-f_{2})I_{2}+f_{1}I_{1}\right]\\ \;-\beta_{1}\frac{f_{2}S_{2}}{f_{2}N_{2}+(1-f_{1})N_{1}}\left[(1-f_{1})I_{1}+f_{2}I_{2}\right]\\ \;+\sigma R_{2}-(1-\nu_{1})Q\frac{S_{2}}{S_{2}+R_{2}} \end{array}$$

(8) $$\begin{array}{l} \frac{\text{d}I_{2}}{\text{d}t}=\beta_{2}\frac{(1-f_{2})S_{2}}{(1-f_{2})N_{2}+f_{1}N_{1}}\left[(1-f_{2})I_{2}+f_{1}I_{1}\right]\\ \;+\beta_{1}\frac{f_{2}S_{2}}{f_{2}N_{2}+(1-f_{1})N_{1}}\left[(1-f_{1})I_{1}+f_{2}I_{2}\right]\\ \;-\gamma I_{2}-\alpha I_{2}. \end{array}$$

(9) $$\frac{\text{d}R_{2}}{\text{d}t}=\gamma I_{2}-\sigma R_{2}-(1-\nu_{1})Q\frac{R_{2}}{S_{2}+R_{2}}.$$

(10) $$\frac{\text{d}V_{2}}{\text{d}t}=(1-\nu_{1})Q.$$

and with:

(11) $$N_{2}=S_{2}+I_{2}+R_{2}+V_{2}.$$

There is substantial flexibility in the selection of parameters in our model. Here we restrict the degrees of freedom by holding most parameters fixed, and as given in Table 1. These values correspond to similarities in the size of the two nations, relatively low initial infection rates, and similar death rates. For the analysis presented here, we primarily focus on the effects of changing the extent of travel, by altering *f*_1_ and *f*_2_, alternative transmission rates β_1_ and β_2_, and importantly the impacts of different time-evolving policies for vaccine sharing, as defined by ν_1_(*t*).

### 2.2. Provision of Vaccines and Their Sharing

We assume that at time *t* = 0 a vaccine becomes available, and a capability exists in nation one to mass produce it from then onwards. The production rate for *t* ≥ 0 is *Q* (vaccines day^−1^). The first nation then has a choice, which might evolve in time, as to the fraction ν_1_(*t*) of vaccines to keep for its own country rather than offering to the second country. The total number vaccinated at time *t* is a simple integration in time of Equations (4) and (10):

(12) $$V_{1}(t)=\int_{0}^{t}\nu_{1}(t^{\prime })Q\text{d}t^{\prime }\;V_{2}(t)=\int_{0}^{t}\left[1-\nu_{1}(t^{\prime })\right]\;Q\text{d}t^{\prime }.$$

Reaching a time when *V*_1_ = *N*_1_ causes ν_1_ ≡ 0 for all times after. This situation is where nation one becomes fully vaccinated, and all vaccines are made available for nation two thereafter. Similarly, if *V*_2_ = *N*_2_, then ν_1_ ≡ 1 for times after. At time *t* = τ (days), then everyone is vaccinated in both nations, i.e., the first time when both *V*_1_ = *N*_1_ and *V*_2_ = *N*_2_. At *t* = τ we stop the simulations and total deaths in nation one, *D*_1_(τ), is noted. For the values of Table 1, then τ ~ 100 days.

Many strategies can be envisaged for vaccine provision, and here we initially search for those that minimise the total number of COVID-19 related deaths for the first nation. That is we look for vaccine sharing pathways, ν_1_(*t*), that satisfy or get near to satisfying, the condition:

(13) $$\text{min }D_{1}(\tau ).$$

Two approaches to searching for optimum solutions are possible. The first is noting that the solution of Equations (1)–(4) and (7)–(10) and discovery of the path ν_1_(*t*) that satisfies Equation (13) is a formal problem in optimal control. This requires derivation of the adjoint to the governing equations, following the optimisation approach of Pontryagin's Maximum Principle (20), and subsequent calculation of solution for ν_1_(*t*), while also satisfying any constraints. Such constraints include that 0 ≤ ν _1_(*t*) ≤ 1, along with further constraints that ensure physical realism e.g., that all *S, I, R*, and *V* values are positive, and *V* ≤ *N*. The second approach is to instead iterate over possible pathways in ν_1_(*t*), subject to the same constraints, and determine for each the *D*_1_(τ) value. This latter approach is far less elegant, and cannot guarantee an overall minimum solution is found. However, there are some advantages. The iterative approach may bring more intuition as to which solutions are particularly sub-optimal, and is easier to implement when there is a necessity of speed in understanding a research problem of concern. Furthermore, a single minimum solution does not necessarily represent an enactable strategy, and of more practical use can be an understanding of the potential outcomes of a range of vaccination strategies. We adopt the second numerical approach, and in particular consider changes to ν_1_ at discrete intervals, which may reflect how policy is enforced.

## 3. Numerical Results

### 3.1. Effect of Delay Before Vaccine Sharing

**Figure 1:**
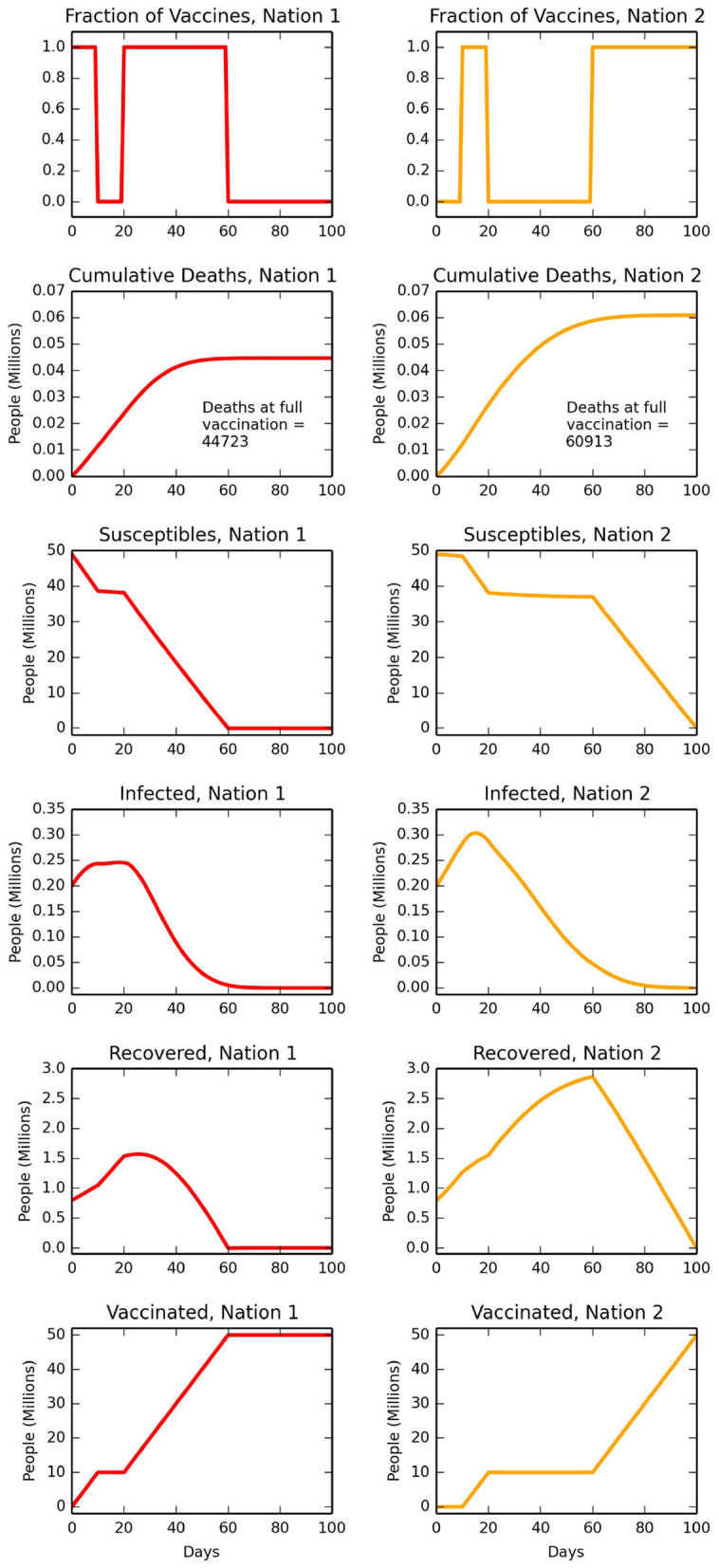
Calculations with parameters and initial conditions as given in Table 1, and including for extensive travel with *f*_1_ = *f*_2_ = 0.2. The left-hand column are time-evolving projections for nation one and that has developed the vaccine, and right-hand column are for nation two. After 20% of the population of nation one are vaccinated, the vaccine is instead used exclusively in nation two. In nation two, 20% of that population is then vaccinated, before returning to fully vaccinate nation one, and then on to fully vaccinate nation two. The top row presents these vaccine sharing decisions, of ν_1_ and 1 − ν_1_. For this scenario, the second row is cumulative deaths (*D*_1_ and *D*_2_), and with annotations of final death count when both countries are fully vaccinated. The next rows are, respectively, the numbers of people susceptible to COVID-19 (*S*_1_ and *S*_2_), infected (*I*_1_ and *I*_2_), recovered (*R*_1_ and *R*_2_) and vaccinated (*V*_1_ and *V*_2_). For each row, the vertical scales are identical to allow comparison between nation one and nation two.

We start by considering a policy where nation one retains all vaccines until a threshold, *X* (%) of its population are vaccinated. At that stage, all further vaccines are given to nation two until they too have *X%* of their population inoculated. Following this, all vaccines are used again in nation one until everyone is vaccinated, after which all further vaccines are given to nation two. We present numerical calculations for *X* = 20% in Figure 1. In Figure 1, the left-hand side panels are time-evolving quantities for nation one, and the right-hand side for nation two. The top row shows this policy choice regarding vaccine sharing (i.e., *X* = 20%), and the second row is cumulative deaths. This policy results in ~25% fewer deaths in the first nation (annotations in second row in Figure 1) as calculated at *t* = τ when both nations are fully vaccinated. In the next four rows, left column are the solutions to Equations (1)–(4), and right column to Equations (7)–(10). In many circumstances, it is the projected number of infected people (row four of Figure 1) that is of most interest to health planners, who need to know if the number of severely ill people may exceed hospital or intensive care unit capacity. The setting of *f*_1_ = *f*_2_ = 0.2 (Table 1) corresponds to extensive inter-nation levels of travel, and this large value is taken to provide an outer bound in our analysis. Some travel exchanges have historically been especially large, including for instance, tourists to key European countries in summer months, although these will be from multiple other nations.

**Figure 2:**
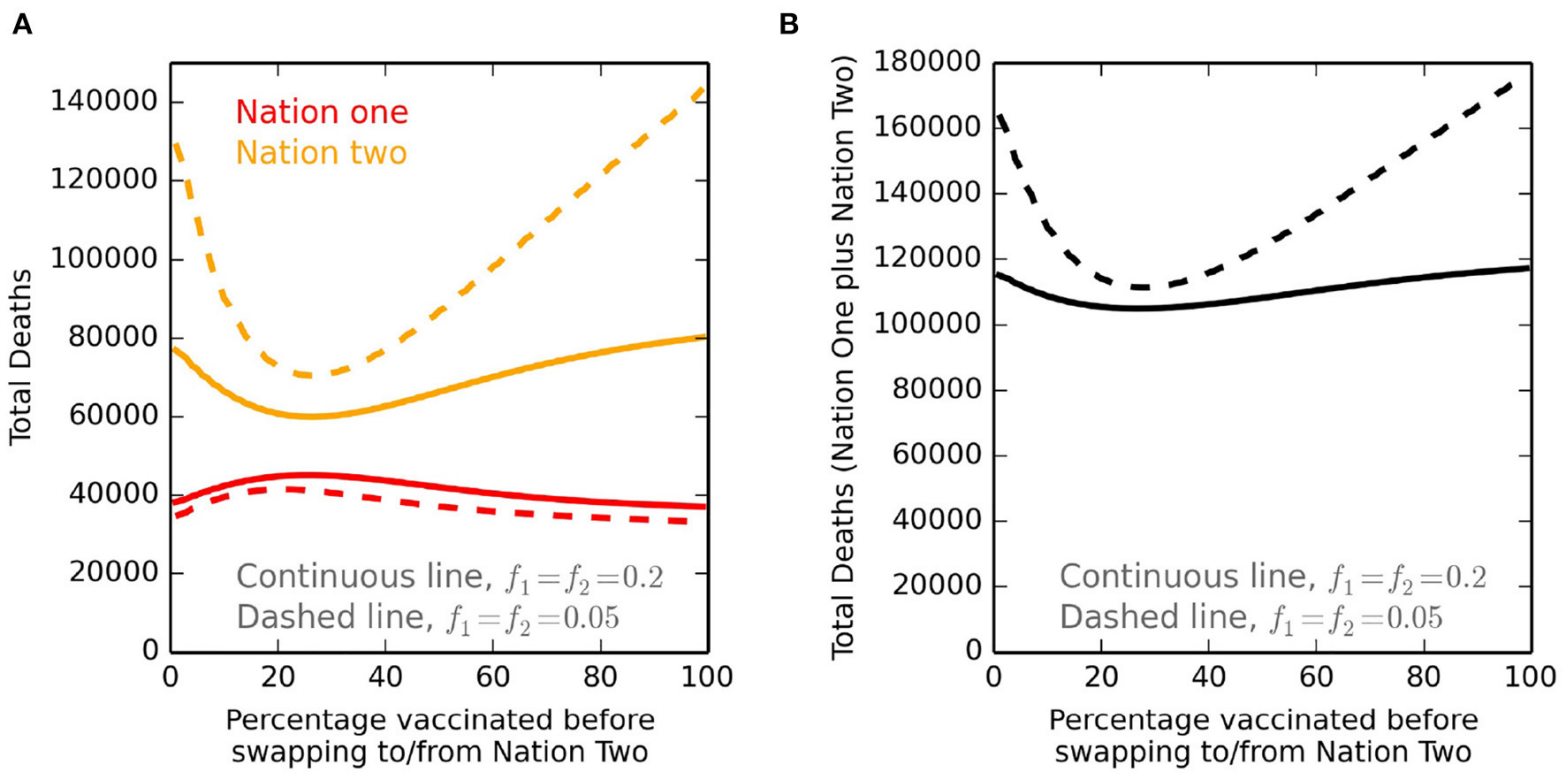
Deaths for different *X* values defining vaccine sharing. **(A)** Total deaths for nation one (red) and nation two (orange), at the time of full vaccination of both nations. The vaccine sharing policy is the same as that presented in Figure 1, except that here the percentage of nation one vaccinated before sharing, *X*, is tested across all percentage values. Hence, for extensive travel exchange with *f*_1_ = *f*_2_ = 0.2 and with *X* = 20%, the values shown are identical to the annotated total deaths in the second row of Figure 1. The continuous lines are for *f*_1_ = *f*_2_ = 0.2, and the dashed lines are for less travel exchange with *f*_1_ = *f*_2_ = 0.05. All other parameters are as given in Table 1. **(B)** Shows the total deaths, and so is the addition of the nation one and nation two deaths of **(A)**.

We repeat the calculations in Figure 1, for all vaccine threshold *X* values, and calculate total deaths in nation one and nation two (Figure 2). COVID-19 related deaths, marked as annotations in the second row of Figure 1, are identical to those at *X* = 20% (Figure 2; continuous lines). Deaths for lower travel between countries, with *f*_1_ = *f*_2_ = 0.05, are also calculated (Figure 2; dashed lines). The curve minimums for nation two (Figure 2A) are a consequence of our sharing framework. For low *X* values, although nation two receives vaccines quickly after *X%* of nation one are inoculated, this only then vaccinates a small percentage *X* of nation two, before nation one continues its immunisation programme. High *X* values cause a substantial time to pass before nation two can start a vaccination programme. Both approaches cause higher deaths compared to the minimum for nation two. For nation one, there is relatively little variation in total deaths, irrespective of the choice of the *X* value. Total deaths in the donor country (nation one) are lowest when the country does not share vaccines (*X* = 0% and *X* = 100%), and peak when the switch of vaccine from donor to recipient country occurs after ~22% of the donor population is vaccinated. Lower travel exchanges (i.e., *f*_1_ = *f*_2_ = 0.05) have a protective influence on nation one, due to fewer imported infections as well as fewer infections by citizens of nation one when overseas. The effect is the opposite for nation two, as the lower amount of travel means a smaller exchange of more infected people from that nation are replaced with those from nation one who are less infected. In addition, lower exchanges from nation two to nation one, which has fewer infections, provides less protection as a smaller number of nation two susceptible people have travelled. Critically, for both sets of *f* values, is that the minimum deaths for nation one correspond to no initial sharing (i.e., at *X* = 100%). Hence for our parameter values, gains by vaccinating people in nation two to lower imported infections are outweighed by no initial sharing, and thus vaccinating all of nation one as fast as possible. The sharing policy by nation one has a much larger impact on deaths in nation two, raising an important ethical issue. If nation one only shares vaccines after all its own citizens have first received it (i.e., *X* → 100%), then nation two has an especially high total number of deaths.

A particular interpretation of the minimum of nation two deaths (Figure 2A) is as follows. For the parameter values reported in Table 1, the basic reproduction number is *R*_0_ = β/γ = 1.25. A very early assessment of the COVID-19 basic reproduction number (21) suggests it to have a much higher value, citing a range of 2.24–3.58, and so implicit in our transmission values is that they are for a later period with social distancing measures in place. Our value of 1.25 suggests that if more than 20% of the population of nation two are vaccinated (achieved in the first phase of vaccine policy if *X* > 10%), then this would make the effective reproduction number, *R* (22), fall below unity. After this, besides the beneficial effects of vaccination, infection numbers would also fall by their own accord achieving what is sometimes referred to as “herd immunity.” Although our sharing scenario is slightly contrived (e.g., top row of Figure 1), for the values presented in Table 1, the minimum for nation two deaths in Figure 2 implies two key features. Giving away enough vaccines such that it allows a nation to have an effective reproduction number substantially below unity will save many lives. However, giving away many more vaccines, but waiting a longer period beforehand will result in more deaths for nation two. For both *f* values (0.2 and 0.05), we show the combined number of deaths for nation one and nation two together (Figure 2B). The minimum number of overall deaths is with an *X* sharing threshold of order 20–25%.

### 3.2. Additional Sensitivity Calculations

Reinfection remains a major uncertainty for the COVID-19 illness (23). Hence, as a factorial experiment, we consider potential non-zero values for immunity waning, σ, after illness. Seow et al. (24) report that acute immunity wanes as expected, but longer immunity from immunoglobulin antobodies can last beyond 94 days. Meanwhile, Dan et al. (25) find that the percentage of subjects seropositive for spike immunoglobulin, at 6–8 months post onset of symptoms, was 90%. For our period of 100 days of simulation, this may be a loss of around 5%, which can be approximated as σ = 0.0005. We repeat the calculations leading to Figure 2, but with this new non-zero value of σ (Supplementary Figure 1). As might be expected, with this relatively low reported loss of immunity, deaths (Supplementary Figure 1) are almost indistinguishable from those for complete immunity (Figure 2).

An additional extension of our analysis is to account for vaccines that do not have 100% efficacy. If 0 ≤ *e* ≤ 1 is efficacy, with for instance a value of *e* = 0.8 corresponding to 80% effectiveness, then an easy amendment to our model is to replace every term *Q* in all equations with *eQ*. This very simplistic characterisation of efficacy would mean that *V* remains the number of vaccinated people, but it only now includes those with full protection. Hence for *e* < 1, the infected, susceptible and recovered groups would be larger compared to if *e* = 1. This alteration is valid where the aim of the model structure is to provide a basic estimate of the number of COVID-19 related deaths. However, in more complex model structures, for instance accounting for different less restricted behaviour by people who are vaccinated, then it may require an additional distinct group for those who have received a vaccine but remain susceptible. As a sensitivity study, we perform the simulations presented in Figure 2 but with *Q* replaced by *eQ* and with *e* = 0.8 (Supplementary Figure 2). We find that the main features of Supplementary Figure 2 are similar to those of Figure 2, but the number of deaths for nations one and two, for each *X* value, are much higher. Our elementary description of efficacy implies that a fraction *e* of those receiving a vaccine cannot be infected. However, reported vaccine efficacies may involve a more subtle definition. In particular a vaccine may be regarded as effective for a fraction *e* of those inoculated if many in that fraction still get infected but the implications are avoidance of serious illness or death. To model this requires a more complex framework with, for instance, an additional infected group of people who have been vaccinated *I*_*v*_, but for whom the fatality rate α_*v*_ (day^−1^) is much smaller than the non-vaccinated value α. Models are emerging that sub-compartmentalise the group infected by COVID19 [e.g., (26)]. An early assessment of the Oxford AstraZeneca vaccine suggests it to be 70% effective (27) and the Pfizer vaccine is reported as being 95% effective (28).

Waning of vaccine immunity can also be accounted for as a further extension of our analysis. In the analysis of vaccine efficacy, characterised by parameter *e*, this corresponds to a fraction, (1 − *e*), of people for whom the vaccine does not work from the outset, but all others receiving it have permanent immunity. To instead model decreasing vaccine immunity, we introduce a daily fraction of those who have received a vaccine, but lose immunity, defined by parameter σ_*V*_ (day^−1^). We modify the nation one vaccine group to account for the lowering of immunity, by adding an extra loss term of −σ_*V*_*V*_1_ to the right hand side of Equation (4). A balancing gain term of +σ_*V*_*V*_1_ is added to the susceptible group *S*_1_, given by Equation (1). Similar changes can be made to the nation two equations for *V*_2_ and *S*_2_. We again repeat the format of the calculations leading to Figure 2, but now including this effect in both nations. Here we imagine the pessimistic scenario whereby new variants cause vaccine immunity loss over a period of 6 months, suggesting σ_*V*_ of order 1/180. Hence we set σ_*V*_ = 0.005 (but *e* = 1), and as expected, this results in more deaths (up to 20,000) based on our parameters (Supplementary Figure 3). Our vaccine rate *Q* and population sizes are such that by day 100, everyone will have received a vaccine. In the circumstances of complete vaccine efficacy (*e* = 1) and permanent immunity (σ_*V*_ = 0), then deaths after our modelled day 100 will be low. However, with low efficacy or loss of immunity, and in the absence of emerging and more effective vaccines, then death rates will remain high after day 100, adding to the totals shown in our diagrams. If the strength of waning immunity is similar for both vaccinated people, and for those in the recovered group after having been ill with COVID-19, then we simulate this with non-zero values for both σ and σ_*v*_. Simulations in Supplementary Figure 4 are identical to those of Supplementary Figure 3, except that now σ is also non-zero, and with σ = σ_*v*_ = 0.005. As expected, this creates a further rise in the number of projected deaths. However, this additional increase through both effects, compared to only σ_*v*_ set as non-zero (Supplementary Figure 4 vs. Supplementary Figure 3), is less than that from introducing the waning vaccine immunity effect only (Supplementary Figure 3 vs. Supplementary Figure 2).

We also consider the impact of initial conditions, and in particular if one nation has substantially more infections at the start of vaccine program. Different infection levels will relate to the previous history of lockdown and social distancing measures within nations. The number of infections at any given time will also depend on the previous timecourse of the index cases, which are the initial people who introduce the virus to a nation (sometimes called “patients zero”). We instead first set nation one to have *I*_1_(0) = 0.5 × 10^6^ initial infections, rather than the lower value of Table 1, and also lower *S*_1_(0) by 0.3 × 10^6^ accordingly. We repeat the calculations leading to Figure 2, but with the new initial condition (Supplementary Figure 5). Again, the salient features of Figure 2 are retained, but of note is that in particular for high exchanges *f*_1_ = *f*_2_ = 0.2, deaths in nation two are much higher, emphasising the effect of exported infections from nation one. We similarly adjust initial infections in nation two, by increasing it to *I*_2_(0) = 0.5 × 10^6^ but keeping all other parameters as for Table 1. For high exchanges of people (*f*_1_ = *f*_2_ = 0.2), now deaths in nation one increase by a large amount due to imported infections through travel (Supplementary Figure 6).

The finding that to minimise overall deaths requires early vaccine sharing by nation one (minimum of curves in Figure 2B) remains valid in our sensitivity studies (Supplementary Figures 1B–6B).

### 3.3. A Broad Range of Vaccine Sharing Policy Options

**Figure 3:**
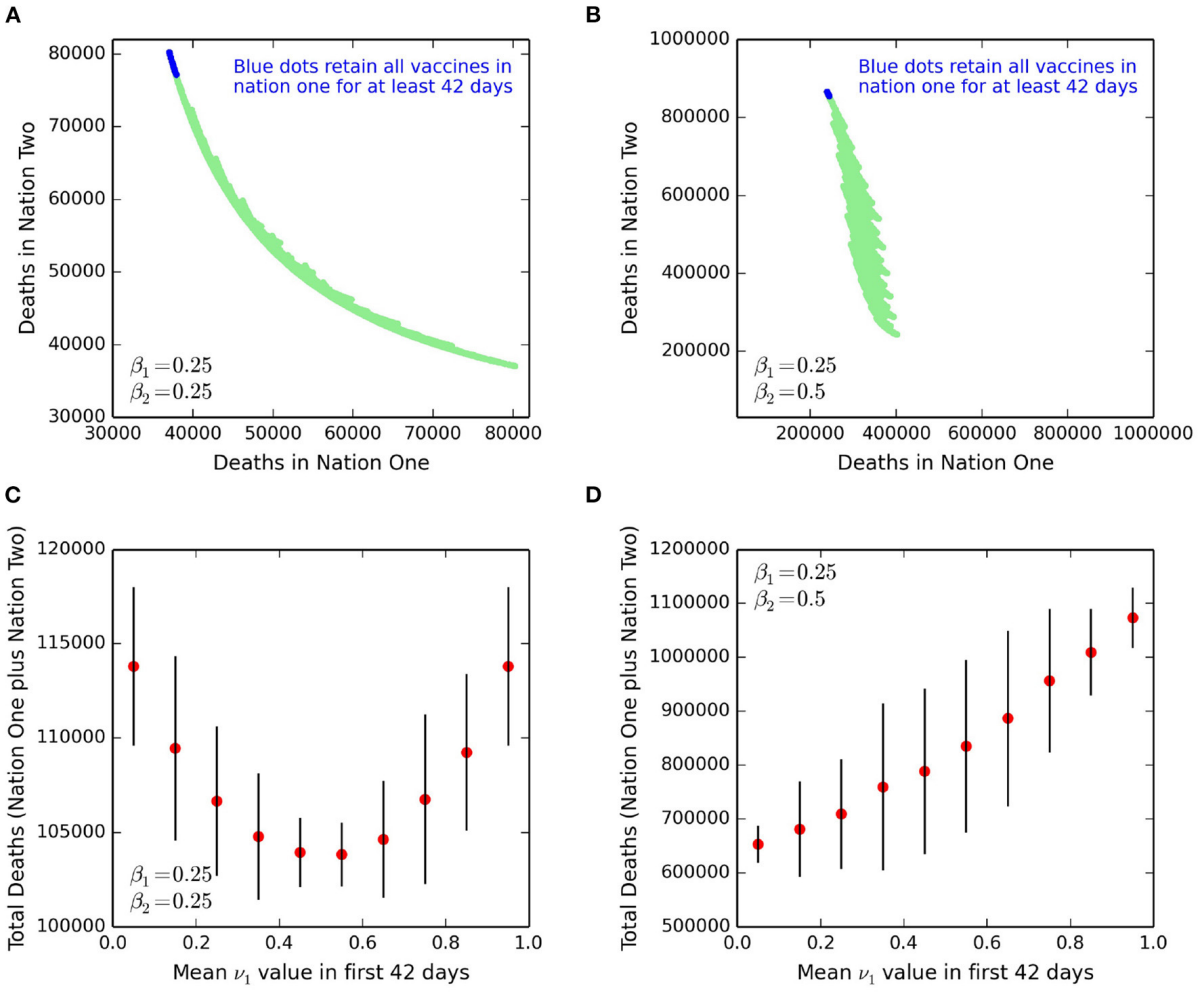
Total deaths in both nation one and nation two at the time of complete vaccination of both nations. Each point in the top row corresponds to a single set of fortnightly policy decisions on vaccine sharing. **(A)** For parameter values and initial conditions presented in Table 1. **(B)** Asymmetric case where virus transmission is more prevalent in nation two, and so with β_2_ = 0.5. In **(B)**, both axes are scaled identically to highlight the different death rates. The blue dots are where nation one retains all vaccines (i.e., ν_1_ ≡ 1) for at least the first three fortnightly periods. In **(C)**, we return to the same parameters and simulations shown in **(A)**, but now show the total deaths (those of nation one plus nation two), and disaggregated by the mean value of vaccine sharing, ν_1_ in the first 42 days. The red dots are the mean values for each “bin” of size 0.1 for ν_1_, and the vertical lines are ± two standard deviations. **(D)** Identical to **(C)**, except for the parameter values of **(B)** i.e., with high virus transmission in nation two.

In a next set of simulations (Figure 3), we scan a much larger range of possible approaches to vaccine sharing. We assume a policy decision is made each fortnight, and when nation one considers changing the value of ν_1_. At days 1, 15, 29, 43, 57, 71, and 85, ν_1_ can be set for the 14 days ahead as either 0.0, 0.2, 0.4, 0.6, 0.8, or 1.0. This policy approach yields 6^7^ potential policy combinations for a given set of parameters. As previously, these sharing options are overridden if one country eventually becomes fully vaccinated, after which all vaccines are used in the other nation. For the set of parameters in Table 1, each policy choice is presented as a dot in Figure 3A. The values (Figure 3A) are the accumulated COVID-19 related deaths, shown for both nations, and up to the time of full vaccination in both nations. The slightly jagged appearance in the spread of solutions is a consequence of the numerical discretisation associated with the fortnightly policy decisions. In Figure 3A, any superimposed line of gradient minus one would correspond to a constant sum of deaths from combining those of both nation one and nation two. Hence the curvature in the plume of points again illustrates a potential issue of ethics. If nation one seeks to minimise its total number of deaths (low values on “x” axis, Figure 3A), then the gradient of points (top left of diagram) has a magnitude larger than unity. As such, every life saved in nation one corresponds to a number greater than unity of lives lost in nation two. Placing a constraint on selected policy options that for the first three fortnights, all vaccines are retained for nation one, corresponds to the blue dots. In similarities to the scenarios presented in Figure 2, such initial high retention levels of vaccines correspond to the lowest cumulative deaths for nation one. This finding suggests again that for the parameters and policy scenarios presented here, the hypothesis that sharing vaccines will lower imported infections and decrease total deaths in nation one cannot be supported when compared to simply vaccinating nation one as fast as possible.

We introduce an asymmetry to the parameters by raising the value of β_2_ for nation two to 0.5 (Figure 3B). If the infectious stage of COVID-19 is 5 days [(15) and our Table 1], then with β_2_ = 0.5, this corresponds to a *R*_0_ value between two and three. This scenario, whereby transmission occurs twice as frequently in nation two, provides an outer bound worst case for our simulations. The new calculations of cumulative total deaths in both nations, at the time when everyone is inoculated and for β_1_ = 0.25 and β_2_ = 0.5, are shown in Figure 3B. The higher value of β_2_, as expected, results in the total deaths in nation two to be vastly higher than those for when β_2_ = 0.25. However, of note is that the deaths in nation one also rises by a large amount, confirming the effect of extensive travel (here *f*_1_ = *f*_2_ = 0.2) to and from nation two that has weaker controls on virus transmission. The blue dots in both panels again correspond to where, for the first three fortnights, nation one retains all vaccines (ν_1_ ≡ 1). To aid illustration, the axis range in Figure 3B for both nations is identical, showing the gradient of the plume of points is now even larger. This high gradient implies that any change in sharing policy that saves additional lives in nation one, will correspond to substantially more lives lost in nation two, again raising ethical issues.

To capture more simply the issue of equity, we present the total number of deaths as a function of initial levels of vaccine sharing (Figures 3C,D). The simulations and parameters of Figure 3C are identical to those of Figure 3A, and similarly Figure 3D uses the same projections as Figure 3B. In Figure 3C, a symmetry exists as expected with identical parameters for nation one and nation two, and so the minimum total deaths are achieved when a half of vaccines are given by nation one to nation two. Figure 3D illustrates that if we model nation two as having a higher transmission rate, then the minimum number of total deaths is when nation one gives away most or all of their vaccines following the start of their production.

## 4. Discussion and Conclusions

### 4.1. General Findings

We have investigated the role of different policies of vaccine sharing on managing COVID-19 infections. Our equation set is designed to model the extent of SARS-CoV-2 virus transmission, during the period when a vaccine is verified as safe and its mass production and distribution starts. Two nations are considered (“one” and “two”), the first of which has discovered and is making a vaccine, and where there is extensive travel between the two countries. We create a model framework to ask whether given travel, it is beneficial for nation one to share vaccines with nation two to lower imported infections? Equations (1)–(4) and (7)–(10), in tandem with the parameters and initial conditions of Table 1, are parameter-sparse, relatively simple, yet capture the main processes needed to address that question. Our headline finding is that for the parameters investigated, despite the risks of imported infection, in order to minimise deaths in nation one the best strategy is for that nation to vaccinate all citizens first before subsequently sharing it. This inference is achieved by iterating numerically over a range of possible sharing strategies. However as expected, lower levels of travel with nation two which has more infections, decreases the number of COVID-19-related deaths in nation one. We recognise the hardship that reduced travel causes, and especially for those with employment in the tourism and hospitality sector. The solutions presented reveal that although some vaccine sharing will likely mean more deaths in nation one, it can cause a disproportionately large saving of lives in nation two. This finding raises obvious ethical issues regarding vaccine distribution. As any particular nation becomes increasingly inoculated, vaccines may then save many more lives by being sent elsewhere.

As a nation starts to achieve a high number of inoculations, reaching herd immunity, then more lives will still be saved with further vaccination. However, at this point, the probability of a life saved per vaccine administered will be higher if it used elsewhere, in a location with little or no vaccine coverage. Vaccine sharing, as a positive externality to infection dynamics, may set up complex issues in control infections and elasticity. Such elasticity is where the prevalence of infection changes the levels of vaccination uptake (29, 30). High elasticity provides self-interested individuals with less incentive to be vaccinated as coverage increases, as they expect to gain from herd immunity. Hence it may become increasingly difficult to minimise the prevalence of infections, and therefore associated deaths, due to COVID-19 in the nation that developed the vaccine. In these circumstances, with a stalling of vaccine uptake, then extensive sharing is likely to achieve further benefits in the first nation by managing overall risks of mortality associated with the virus. Sharing will reduce risks of imported infections where there is substantive travel with other nations.

### 4.2. Caveats

The modelling framework used in this study is not exempt from caveats and limitations. For instance, we have not explored where two nations have markedly different populations and so *S*_1_(0) and *S*_2_(0) are dissimilar. The fractions of nations travelling, given by *f*_1_ and *f*_2_, may also not balance, for instance should one country be a popular tourist destination. Temporary self-quarantining of people after travel, possibly in tandem with raised testing regimes (e.g., at airports), will lower levels of imported infections, and these effects would require adjustment to our equations. In addition, some parameters may not be fixed in time. For instance, the number of vaccines that can be produced per day may grow substantially in time, making *Q* time-dependent. Arguably, any nation seeing the benefits of vaccination causing immunity may choose to simultaneously work to lower transmission rate further (i.e., make β a function of time), as part of a push to completely remove the virus as fast as possible. A further caveat is that our equations do not include any within-country compartmentalisation of populations, noting others conclude that vaccine priority should be for those at greater risk, such as the elderly and the immune-compromised (31, 32). The assumption of complete or high immunity for those who recover from COVID-19 remains an open scientific question. Investigating more extreme parameter ranges or policy options may yet find that the best solution, in terms of cumulative deaths of nation one, is some early vaccine sharing. Our flexible mathematical structure may be applicable at more local scales within countries, to understand different policies for major cities and between which substantial travel occurs. In the other direction, the simulation structure is available to extension to understand interactions between more than two countries.

A further caveat is that we do not account for any fraction of infected individuals who, upon realising they are unwell and suspect (correctly) they have COVID-19, decide to not travel. If *a* is the fraction of infected people who plan to travel, and still travel, then this would likely also include the sizeable number of people with COVID-19 who are asymptomatic. Inclusion of this effect would modify, for instance, Equation (1) for susceptible people in nation one, to instead be as given as Supplementary Equation (1). In the instance where nation two has high infection levels, then the implication for those susceptible in nation one of less travel by unwell people (i.e., by a lower *a* value) in Supplementary Equation (1) is as follows. The first right-hand term of Supplementary Equation (1) will be affected mainly by the last component, with *af*_2_*I*_2_ replacing *f*_2_*I*_2_ in Equation (1). This change implies that fewer infected people will travel from nation two to nation one, lowering imported infections. However, such suppression of travel by this mechanism will have less effect on the second right-hand term of Supplementary Equation (1), which describes the risk of infection by *f*_1_*S*_1_ susceptibles (i.e., non-infected people) in nation one travelling to nation two.

A major ongoing concern of the COVID-19 crisis is the emergence of virus variants (e.g. in Brazil, India, United Kingdom and South Africa), and that might be vaccine resistant, have higher transmission levels, or both. For instance, the Delta variant first found in India may have more vaccine resistance (33). If a new variant becomes the dominant strain, then our framework can accommodate this by different vaccine efficacy *e*, waning vaccine immunity σ_*v*_, and transmission β parameter values. If reducing overall virus infection globally lowers the risk worldwide of new variants of concern emerging, then this relates to vaccine sharing should some policies lower overall infection levels more than others.

More complexity can be added to our simulation framework by configurations that consider variation in transmissibility and susceptibility within the populations of nations. Distinctions may be defined by age-dependent variation in social mixing (34) and mortality, clinically vulnerable vs. non-vulnerable, or variation in existing medical conditions that may partially depend on poverty levels. Specific vaccine rollout plans may also be modelled, such as prioritising vaccinations to frontline health workers who will have much higher levels of interactions with infected individuals. Such variations would require substantial additional parameters (mortality levels and inter-group infection transmission rates) and model compartments, all requiring quantification. More generally, whilst many countries have focussed on vaccinating the eldest first, as they are considered more vulnerable, the COVID-19 crisis raises other issues of inter-generational equity. For instance, younger people may be disproporitionately affected by unemployment, post-COVID-19 national debt burdens, and research is needed to see if they are particularly impacted by mental health issues caused by lockdowns (35). The order of within-country vaccination of groups may affect these factors, for instance, by inoculating some groups faster to enable return to full employment. The issue of elasticity in vaccine uptake is also not included in our equations.

The equations presented and their solution to find a vaccine sharing policy that minimises deaths is amenable to the application of optimal control theory. Such methods have been successful in informing public health strategies regarding the avian influenza pandemic (36), the Chikungunya epidemic (37), and influenza (38). Optimal control has also been used in terms of minimising the cost of vaccine programmes, for human papillomavirus (HPV) (39) and influenza (40), and sometimes in tandem with other disease prevention methods e.g., mosquito control for dengue (41). Optimal control methods are elegant, ultimately the most appropriate mathematically, and provide a level of verification unachieveable by scanning numerically for a solution. We plan to undertake such analyses, solving the governing equations and additionally the adjoint, as required by optimal methods, along with satisfying constraints to ensure physical realism. However, our more fast-track initial “forward-mode” computation approach does have some advantages. We restricted ourselves to discrete periods of time between policy changes, potentially reflecting how decisions are undertaken with regular reviews. The discrete changes could, though, be used to approximate any smooth time-evolving trajectories discovered for ν_1_(*t*) by optimal techniques.

### 4.3. Overall Summary

Our analysis represents two countries of similar size and levels of visitation rates, and where we set transmission rates to values that might reflect the on-going implementation of NPIs such as the use of lockdowns, personal protection equipment, and social distancing. The mass production and use of vaccines is considered to start at the beginning of our simulations. We assume that using a general SIRV (Susceptible, Infected, Recovered, Vaccinated) model is valid to describe the spread of COVID-19 illness. For our selected parameters and range of policy options, we find that reducing travel and keeping all vaccines until full inoculation will minimise COVID-19 related deaths in a nation (nation one) that produces a vaccine. Our initial hypothesis was that when accounting for travel, it is beneficial for nation one to share vaccines with nation two, to lower either imported infections, or infection risk when visiting nation two. For our selected default parameters, this effect appears relatively small. However, the extent of travel affects nation two more, as for example with larger exchanges, people of nation two are more protected when visiting nation one. If either nation has a higher initial infection level at the start of a vaccine program, as expected, travel will cause more deaths in the other nation.

What our calculations do highlight is the strong influence that any vaccine sharing policy has on the total deaths of nation one and two combined. In particular, to minimise deaths overall, nation one needs to offer nation two a substantial number of vaccines, and early on. In some instances, extensive sharing may result in only small increases in deaths in nation one (the vaccine producer), yet save a much larger number of lives in nation two. This finding also remains valid for the sensitivity calculations we performed and report. Early and sizeable vaccine sharing raises an ethical dilemma. Should the government of a nation producing a vaccine make their primary role to inoculate as fast as possible all those who have elected it, or to take a more global perspective, and share earlier on vaccines to save more lives overall? The issue of vaccine sharing and related ethics is likely to require substantial thought in the months ahead. Indeed, as of the beginning of year 2021, there has already been tension between the European Union and the United Kingdom on this matter. Although a very obvious point to make, it is worth reiterating that with the emergence of vaccines, deaths will be minimised by achieving its largest possible mass production. High production levels will most quickly vaccinate the country of its origin, and enable more rapid and substantial sharing internationally. As vaccines are now receiving approval as safe, our model framework can be used in a more operational context, entraining known parameters specific to individual countries.

## Data Availability Statement

The computer model scripts supporting the conclusions of this article will be made available by the authors, without undue reservation. The python computer scripts are also provided, in full, in the Supplementary Material.

## Author Contributions

CH conceived the conceptual framework and designed the associated numerical experiment. CH and TR built the equation set, and their numerical solution. MB provided advice on current COVID-19 understanding. All authors reviewed and contributed to writing the manuscript.

## Conflict of Interest

The authors declare that the research was conducted in the absence of any commercial or financial relationships that could be construed as a potential conflict of interest.

## Publisher's Note

All claims expressed in this article are solely those of the authors and do not necessarily represent those of their affiliated organizations, or those of the publisher, the editors and the reviewers. Any product that may be evaluated in this article, or claim that may be made by its manufacturer, is not guaranteed or endorsed by the publisher.
