# Supplementary material for: Optimal COVID-19 Vaccine Sharing Between Two Nations That Also Have Extensive Travel Exchanges
Source: Front Public Health. 2021 Aug 12;9:633144. doi: 10.3389/fpubh.2021.633144 (PMC8387873; doi:10.3389/fpubh.2021.633144)
Supplement: Supplementary file 1 [file Data_Sheet_1.zip › supplemental_data_zip/python_code/figure1/figure1.pdf]

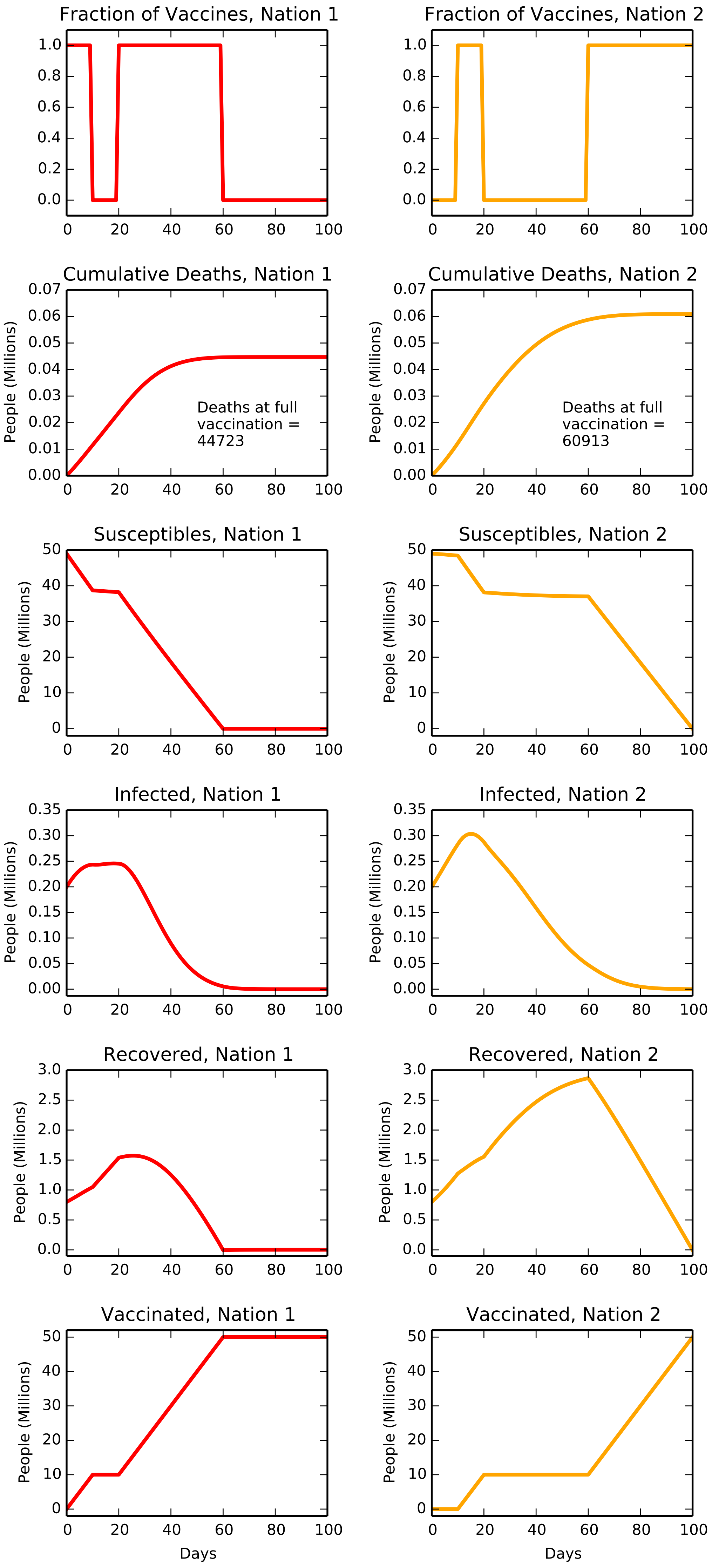
Fraction of Vaccines, Nation 1
Fraction of Vaccines, Nation 2
Cumulative Deaths, Nation 1
Deaths at full vaccination = 44723
Cumulative Deaths, Nation 2
Deaths at full vaccination = 60913
Susceptibles, Nation 1
Susceptibles, Nation 2
Infected, Nation 1
Infected, Nation 2
Recovered, Nation 1
Recovered, Nation 2
Vaccinated, Nation 1
Vaccinated, Nation 2
People (Millions)
Days
