# Supplementary material for: Optimal COVID-19 Vaccine Sharing Between Two Nations That Also Have Extensive Travel Exchanges
Source: Front Public Health. 2021 Aug 12;9:633144. doi: 10.3389/fpubh.2021.633144 (PMC8387873; doi:10.3389/fpubh.2021.633144)
Supplement: Supplementary file 1 [file Data_Sheet_1.zip › supplemental_data_zip/supplementary_information.pdf]

Supplemental Information to paper: "Optimal COVID-19 vaccine sharing between two nations that also have extensive travel exchanges"

Chris Huntingford, Thomas Rawson and Michael B. Bonsall

Accounting for those who are unwell and chose not to travel

A potential modification of Equation (1) is to account for unwell infected people who do not travel. Specifically, this is the time-evolving number of susceptible people in nation one, $S_1(t)$, now accounting for only a fraction a of infected people who intended to travel and still do. This leaves fraction $(1-a)$ who would have travelled, but do not as they are unwell. All variables and parameters are as for Equation (1), with the addition of parameter a. This new Supplementary Equation is as:

$$\begin{aligned}\frac{\mathrm{d}S_1}{\mathrm{d}t} =& -\beta_1 \frac{(1-f_1)S_1}{(1-f_1)[N_1-I_1]+(1-af_1)I_1+f_2[N_2-I_2]+af_2I_2}\left[(1-af_1)I_1+af_2I_2\right] \\ & -\beta_2 \frac{f_1S_1}{f_1[N_1-I_1]+af_1I_1+(1-f_2)[N_2-I_2]+(1-af_2)I_2}\left[(1-af_2)I_2+af_1I_1\right] \\ & +\sigma R_1 - \nu_1 Q\frac{S_1}{S_1+R_1}. \qquad \text{(Supplementary Equation 1)}\end{aligned}$$

Equations (2), (7) and (8) require a similar adjustment.

Additional sensitivity analyses - Supplementary Figures 1-6

As a sensitivity analysis, we repeat Figure 2 in the main paper, but instead with a non-zero value of immunity waning. A value of $\sigma > 0$ corresponds to some loss of immunity. The literature cited in the main paper suggests only small loses of immunity occur, and that we approximate with a value of $\sigma = 0.0005$ day^{-1}. We use this value in a set of simulations that are otherwise identical to those leading to Figure 2, instead giving Supplementary Figure 1 below.

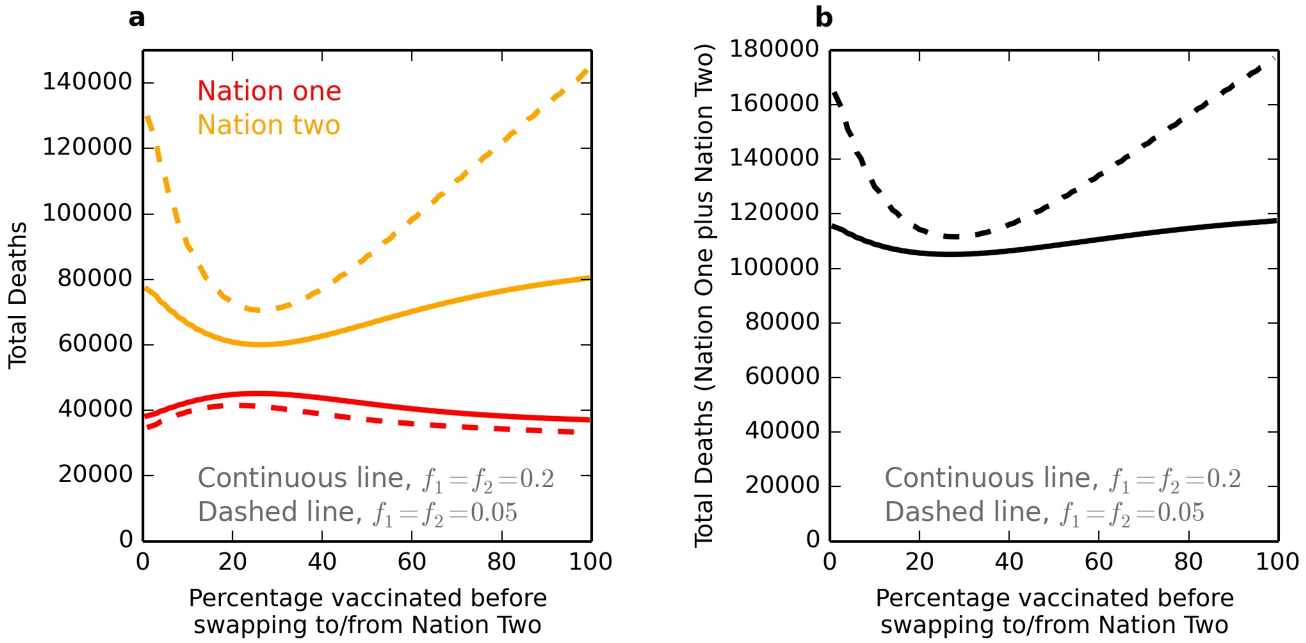

Supplementary Figure 1. Identical diagram to Figure 2, but with incomplete immunity parameterised as $\sigma = 0.0005$ day^{-1}. Curves with $f_1 = f_2 = 0.2$ correspond to extensive travel exchanges between nation one and nation two, and $f_1 = f_2 = 0.05$ are for less travel.

As a further sensitivity analysis, we repeat Figure 2 in the main paper, and now with vaccine efficacy at less than 100%. Instead, we set vaccine efficacy as 80%, corresponding to $\epsilon = 0.8$. This change is in simulations that are otherwise identical to those leading to Figure 2, instead giving Figure S2 below.

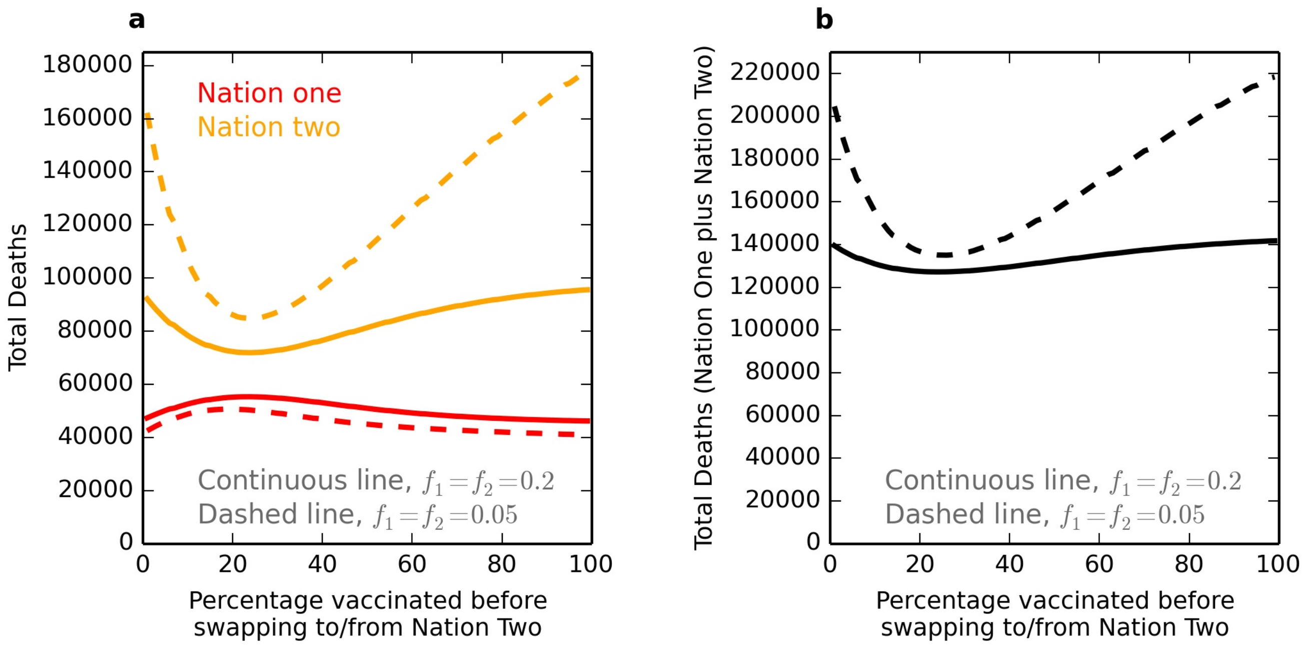

Supplementary Figure 2. Identical diagram to Figure 2, but with vaccine efficacy less than 100%, and instead set at 80% corresponding to $\epsilon = 0.8$. Curves with $f_1 = f_2 = 0.2$ correspond to extensive travel exchanges between nation one and nation two, and $f_1 = f_2 = 0.05$ are for less travel.

Our analysis also considers the effect of vaccines not having permanent immunity. Losing immunity is different to vaccine efficacy at less than 100%. Lower efficacy implies that for some people, the vaccine does not provide protection at any time, whereas for those it does work for, they have indefinite immunity. We characterise the loss of immunity by setting vaccine immunity waning parameter σ_v as taking a non-zero value. A set of simulations are performed with $\sigma_v = 0.005$, but are otherwise identical to those leading to Figure 2. These simulations are presented in Supplementary Figure 3. The analysis leading to Supplementary Figure 3 is extended further to also include simultaneous decreases, in time, of immunity of those who have recovered from COVID-19. We set the rate of immunity loss to be identical between the recovered and vaccinated groups, with $\sigma = \sigma_v = 0.005$, and with the projections as given in Supplementary Figure 4. Unlike in Supplementary Figure 2, the value of $\sigma = 0.05$ is an order of magnitude larger, and may correspond to emerging variants against which a vaccine loses its protection.

We perform calculations for different initial levels of infection. We once again repeat the calculations leading to Figure 2, and now with higher starting infection levels in nation one of $I_1 = 0.5 \times 10^6$ people (and with S_1 lowered slightly to $S_1 = 48.7 \times 10^6$). The resulting simulations are shown in Supplementary Figure 5 below.

Supplementary Figure 6 is similar to Supplementary Figure 5, but instead corresponds to higher initial infection levels for nation two. Now all values are identical to those of Table 1, except that $I_2 = 0.5 \times 10^6$ people (and with S_2 lowered slightly to $S_2 = 48.7 \times 10^6$).

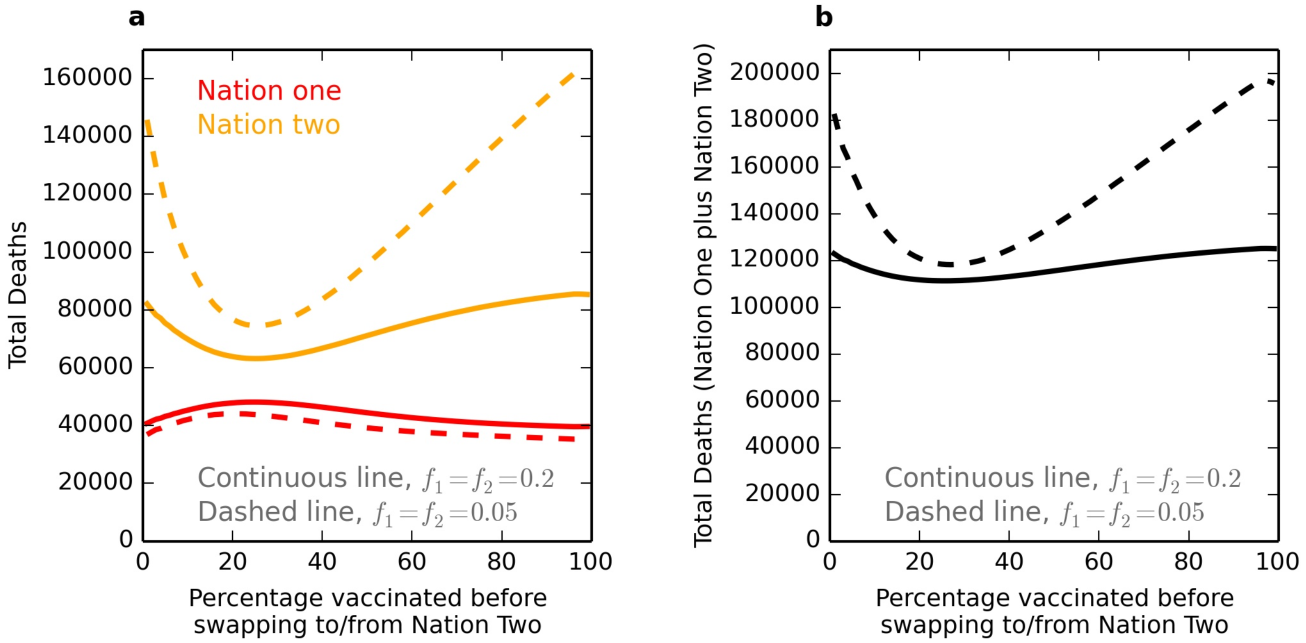

Supplementary Figure 3. Identical diagram to Figure 2, but with vaccines modelled as not having permanent immunity. The simulations have waning vaccine immunity, set as $\sigma_v = 0.005$. Curves with $f_1 = f_2 = 0.2$ correspond to extensive travel exchanges between nation one and nation two, and $f_1 = f_2 = 0.05$ are for less travel.

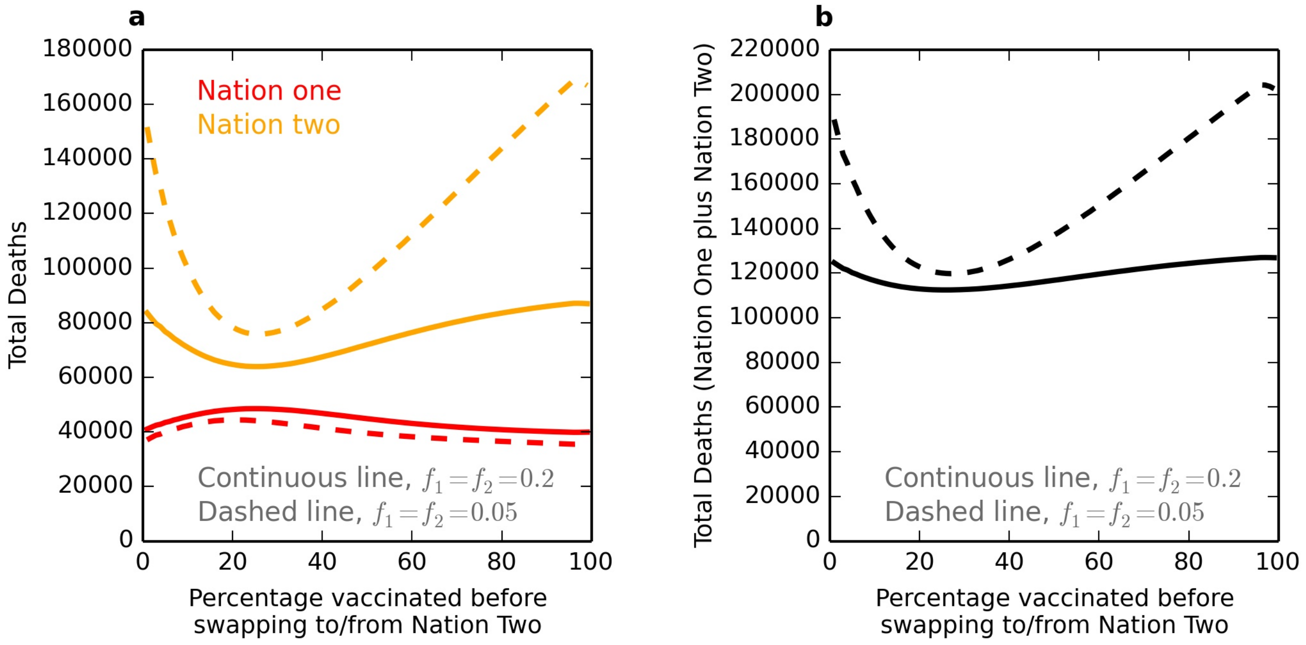

Supplementary Figure 4. Identical diagram to Figure 2, but with both vaccines and those recovered from COVID-19 modelled as not having permanent immunity. The simulations have waning immunities set as $\sigma = \sigma_v = 0.005$. Curves with $f_1 = f_2 = 0.2$ correspond to extensive travel exchanges between nation one and nation two, and $f_1 = f_2 = 0.05$ are for less travel.

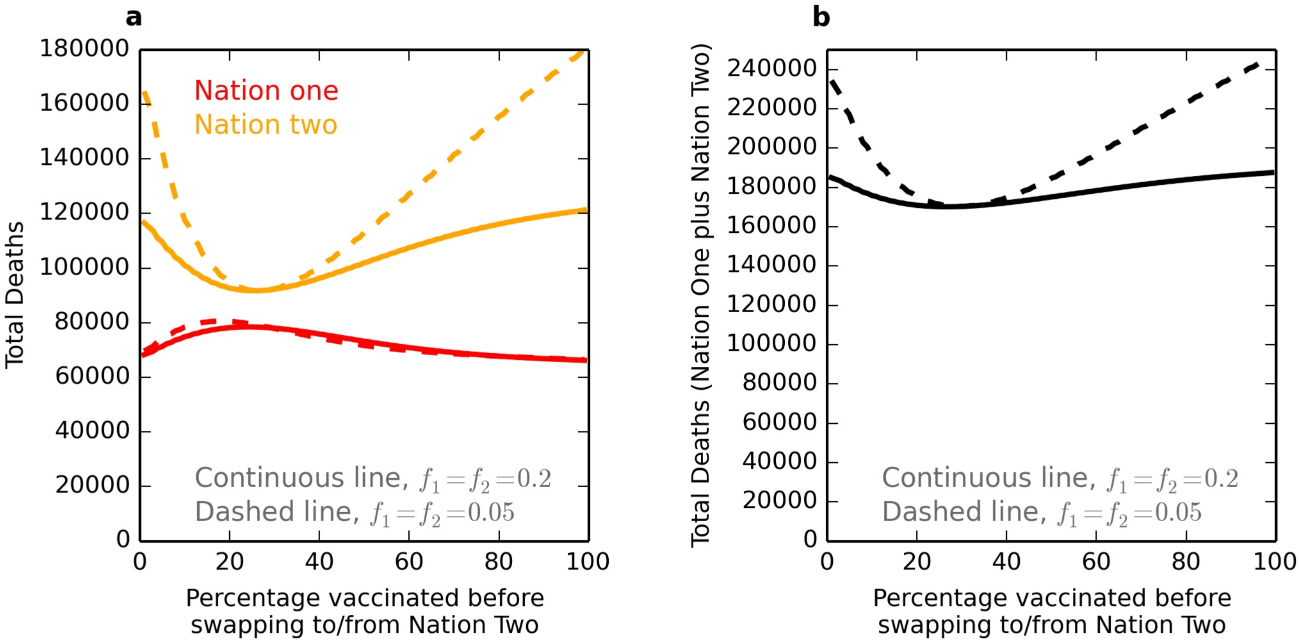

Supplementary Figure 5. Identical diagram to Figure 2, but with initial infections of nation one raised to $I_1 = 0.5 \times 10^6$ people (and initial number of susceptible people lowered by the equivalent amount to $S_1 = 48.7 \times 10^6$). Curves with $f_1 = f_2 = 0.2$ correspond to extensive travel exchanges between nation one and nation two, and $f_1 = f_2 = 0.05$ are for less travel.

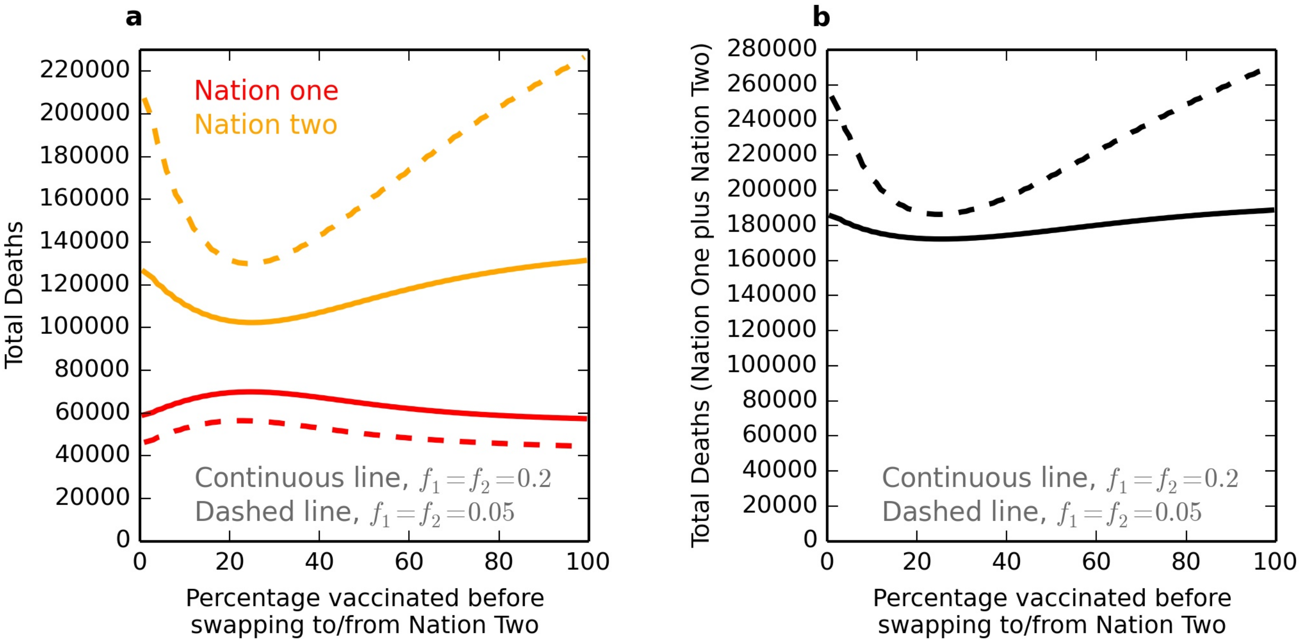

Supplmentary Figure 6. Identical diagram to Figure 2, but with initial infections of nation one raised to $I_2 = 0.5 \times 10^6$ people (and initial number of susceptible people lowered by the equivalent amount to $S_2 = 48.7 \times 10^6$). Curves with $f_1 = f_2 = 0.2$ correspond to extensive travel exchanges between nation one and nation two, and $f_1 = f_2 = 0.05$ are for less travel.
