# Supplementary figures and images for: Optimal COVID-19 Vaccine Sharing Between Two Nations That Also Have Extensive Travel Exchanges
Source: Front Public Health. 2021 Aug 12;9:633144. doi: 10.3389/fpubh.2021.633144 (PMC8387873; doi:10.3389/fpubh.2021.633144)

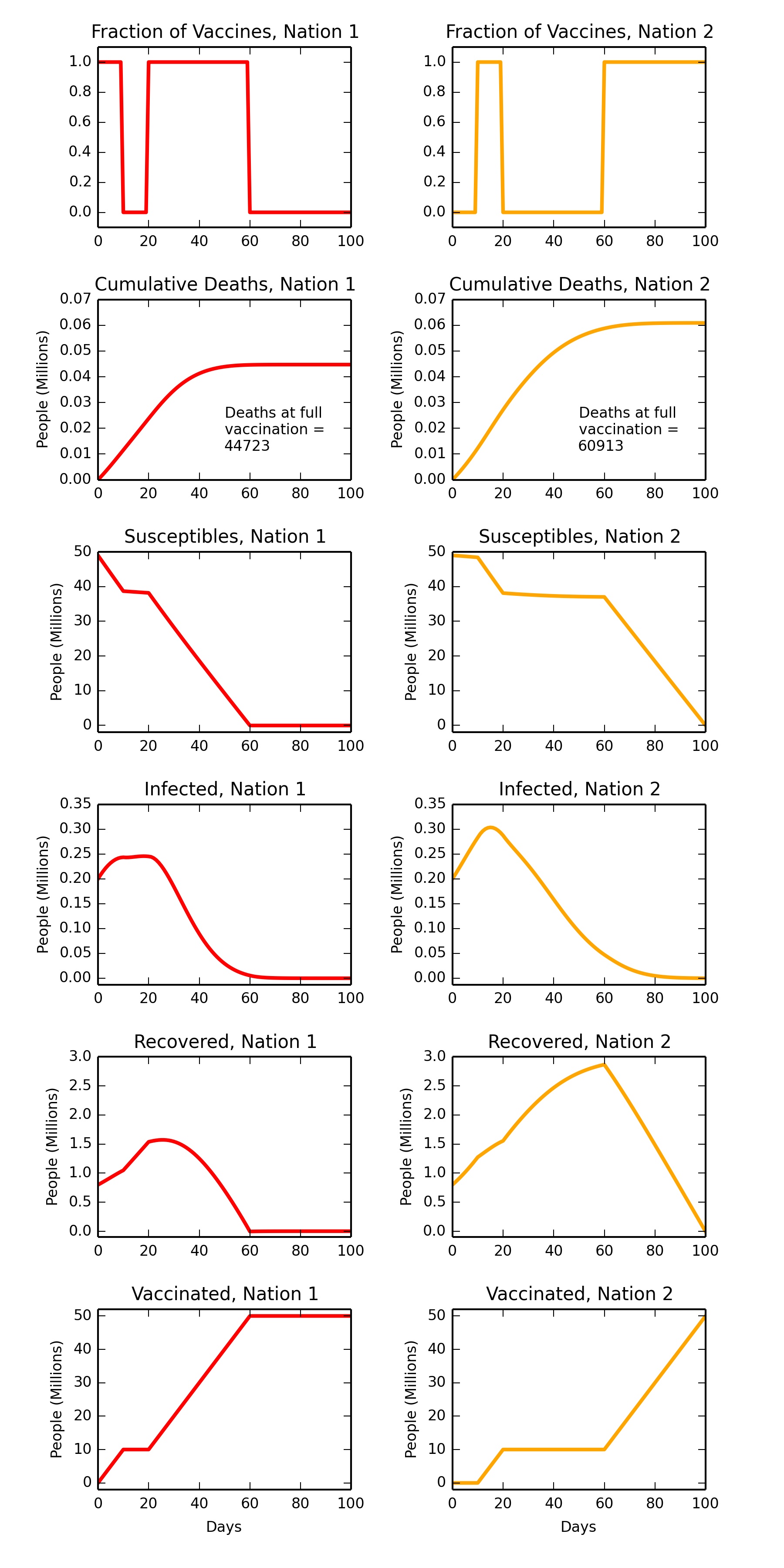

Supplement: Supplementary file 1 [file Data_Sheet_1.zip › supplemental_data_zip/python_code/figure1/figure1.jpg]

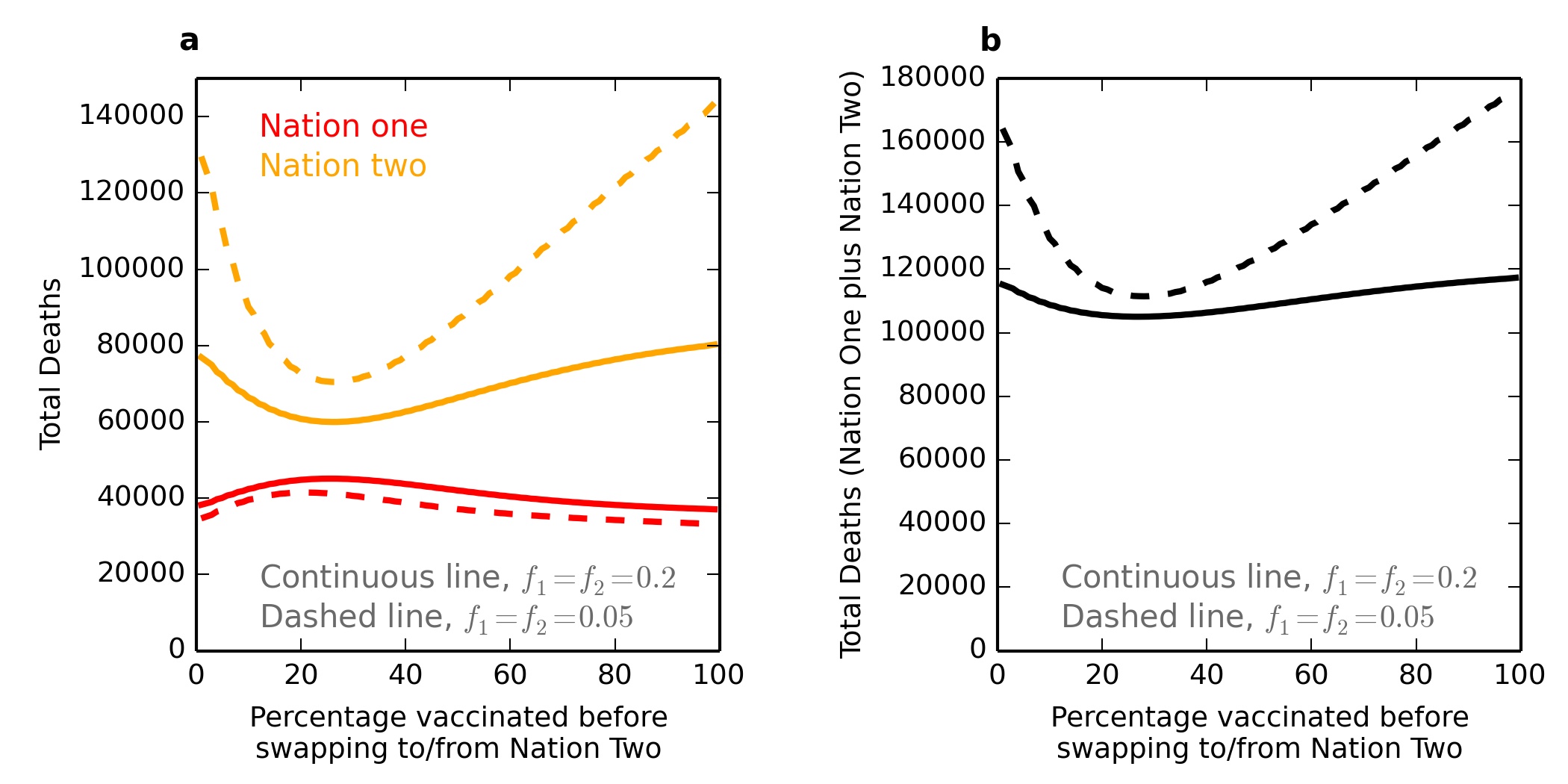

Supplement: Supplementary file 1 [file Data_Sheet_1.zip › supplemental_data_zip/python_code/figure2/figure2.jpg]

**a**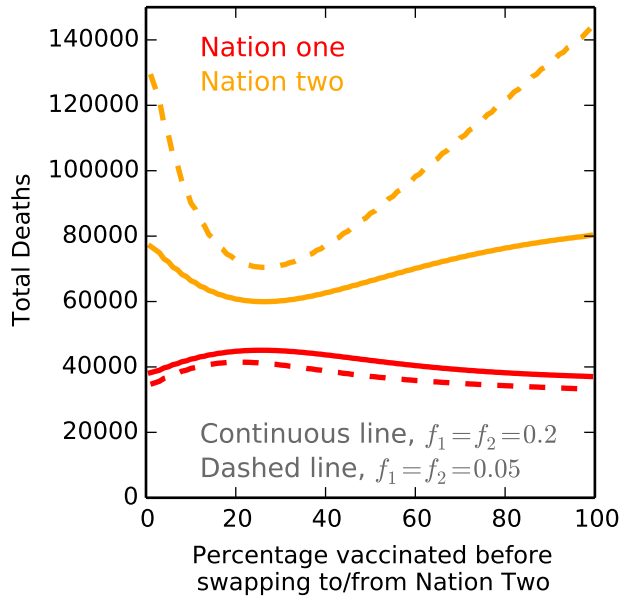**b**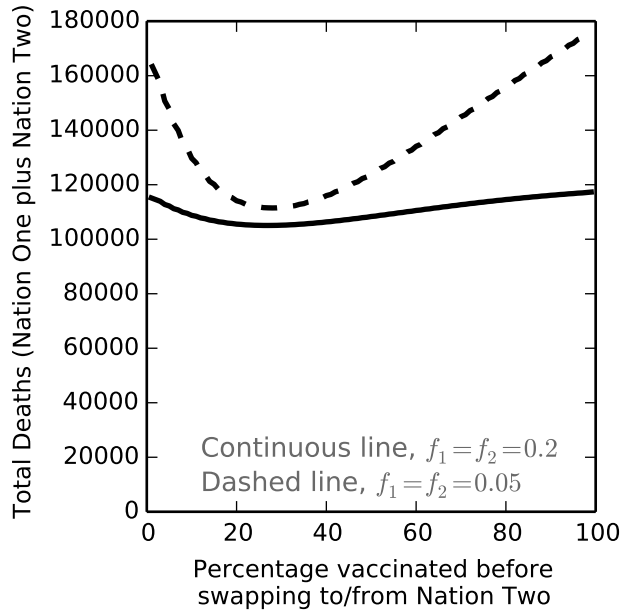

Supplement: Supplementary file 1 [file Data_Sheet_1.zip › supplemental_data_zip/python_code/figure2/figure2.pdf]

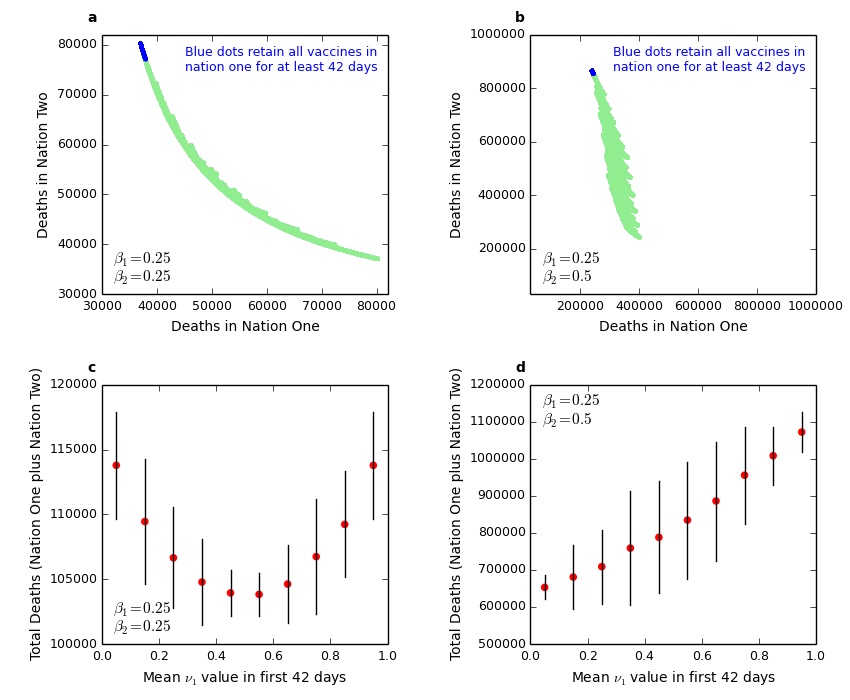

Supplement: Supplementary file 1 [file Data_Sheet_1.zip › supplemental_data_zip/python_code/figure3/figure3.jpg]

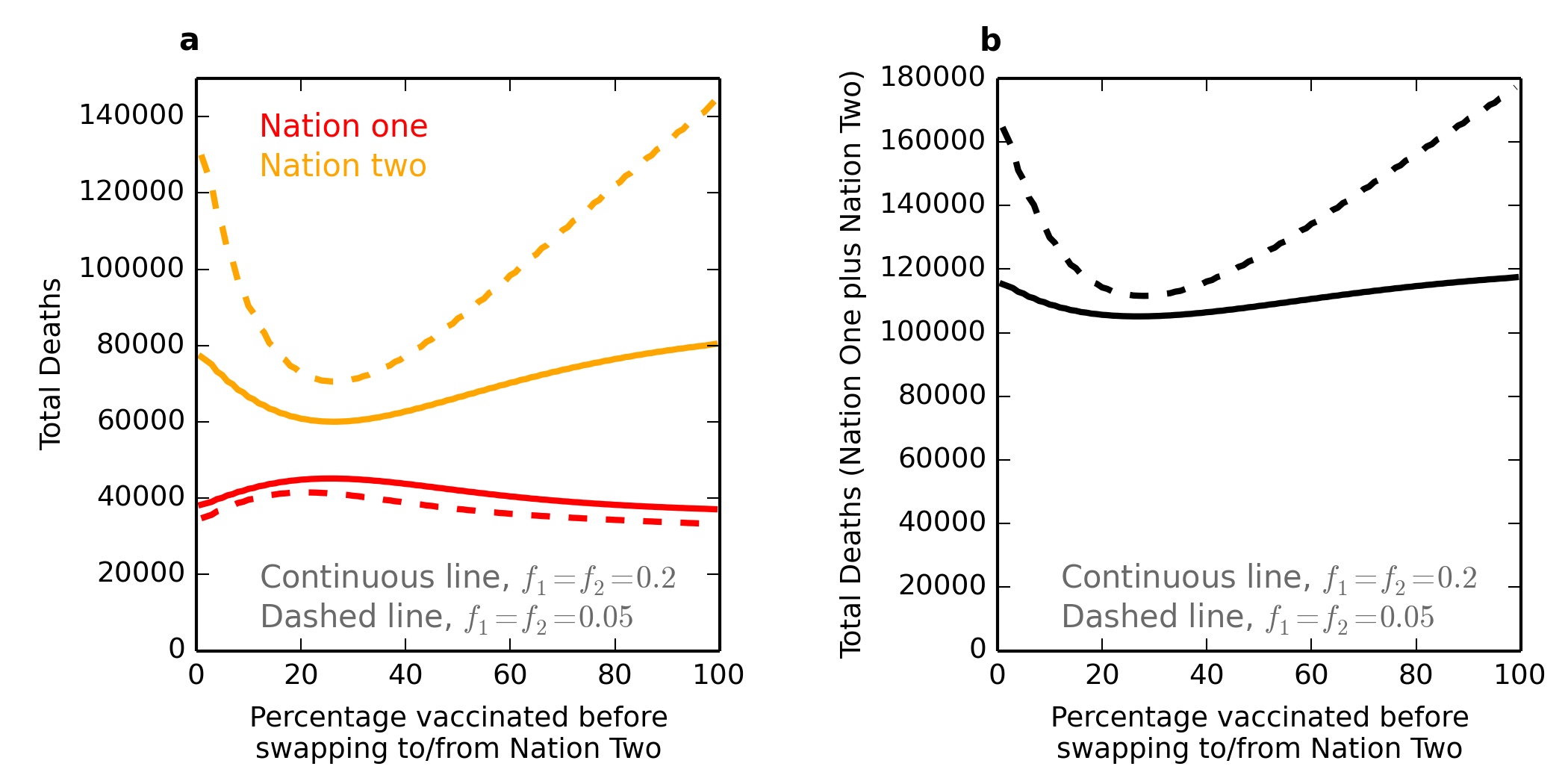

Supplement: Supplementary file 1 [file Data_Sheet_1.zip › supplemental_data_zip/python_code/figureS1/figureS1.jpg]

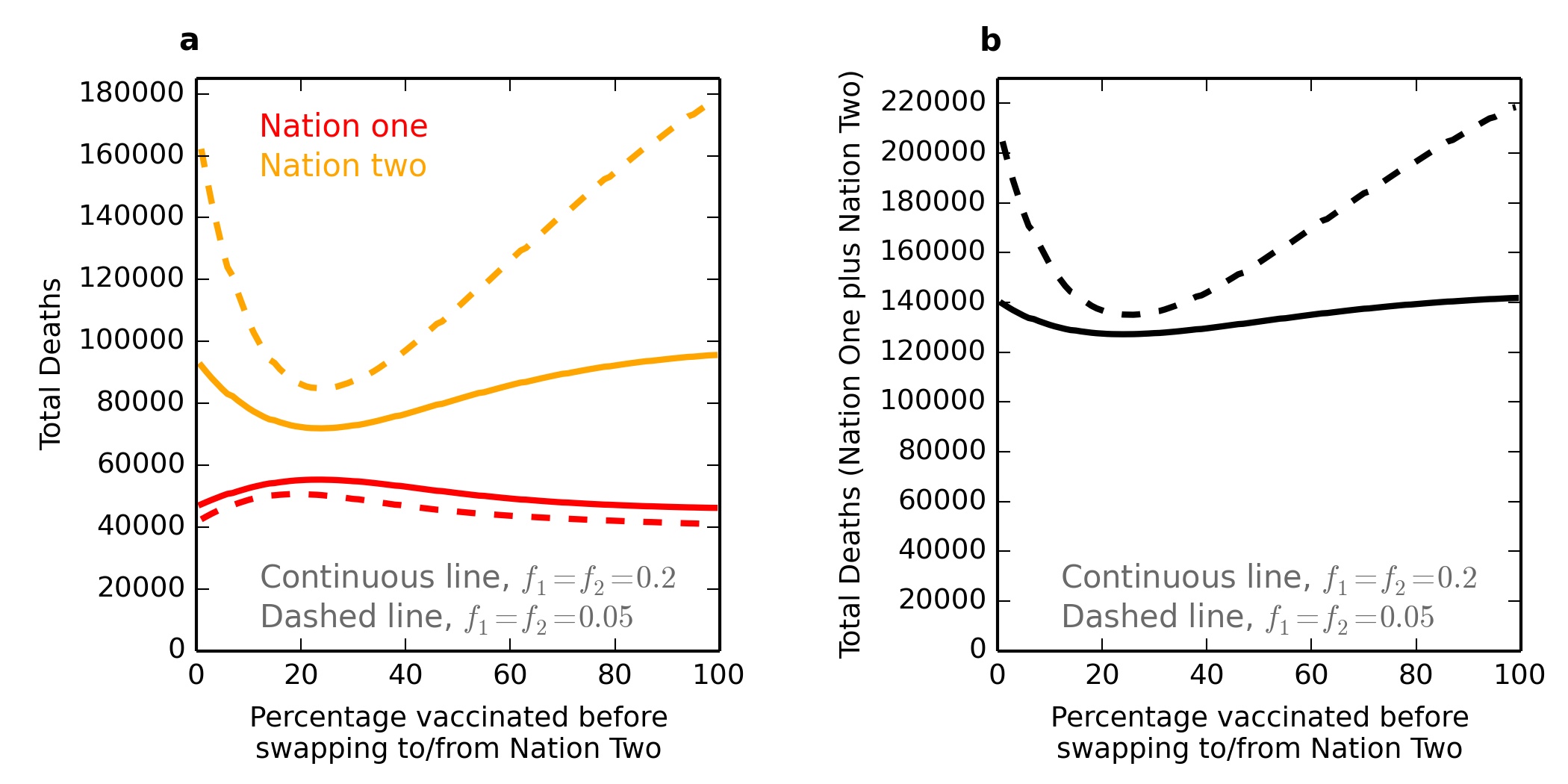

Supplement: Supplementary file 1 [file Data_Sheet_1.zip › supplemental_data_zip/python_code/figureS2/figureS2.jpg]

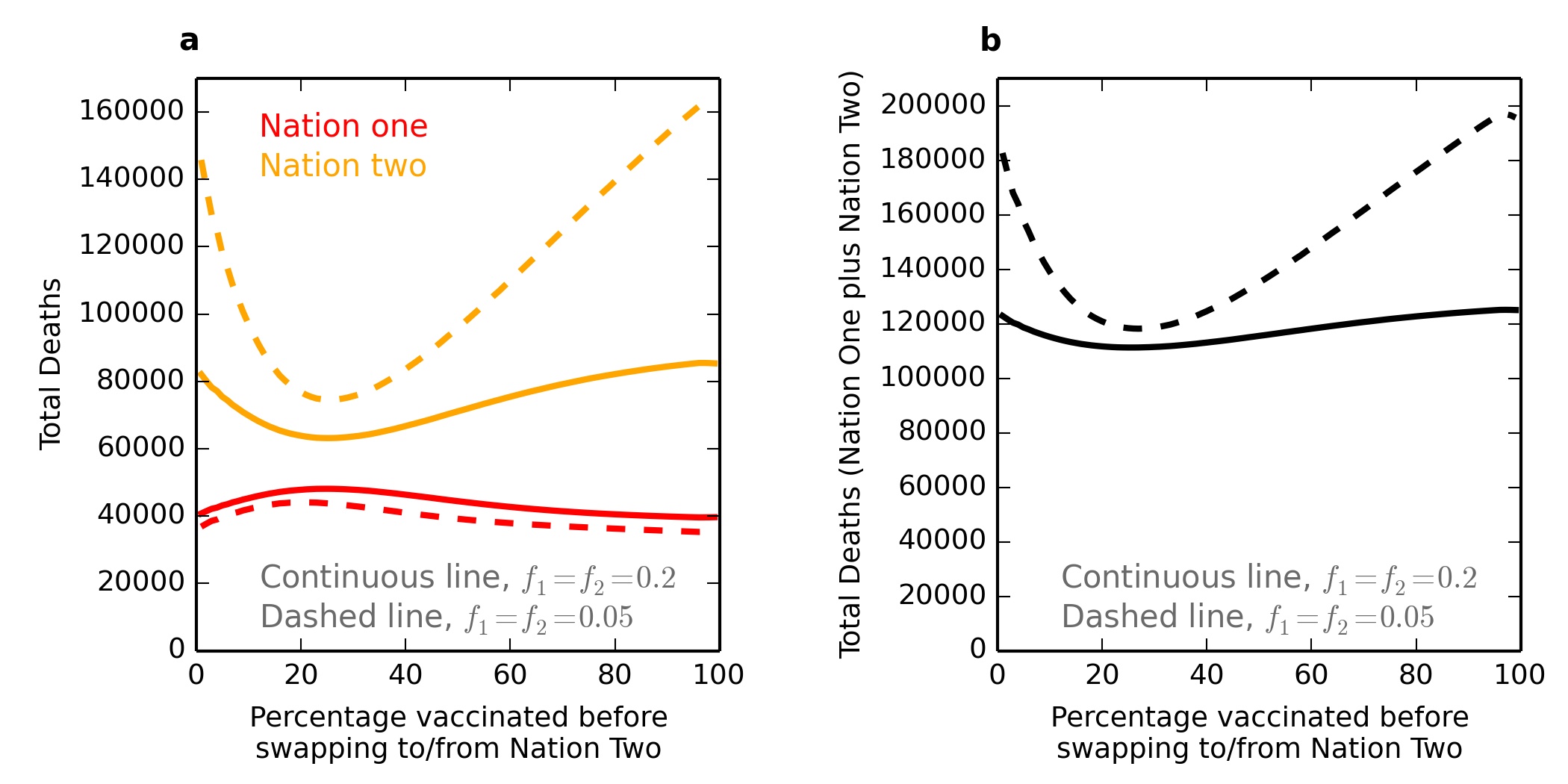

Supplement: Supplementary file 1 [file Data_Sheet_1.zip › supplemental_data_zip/python_code/figureS3/figureS3.jpg]

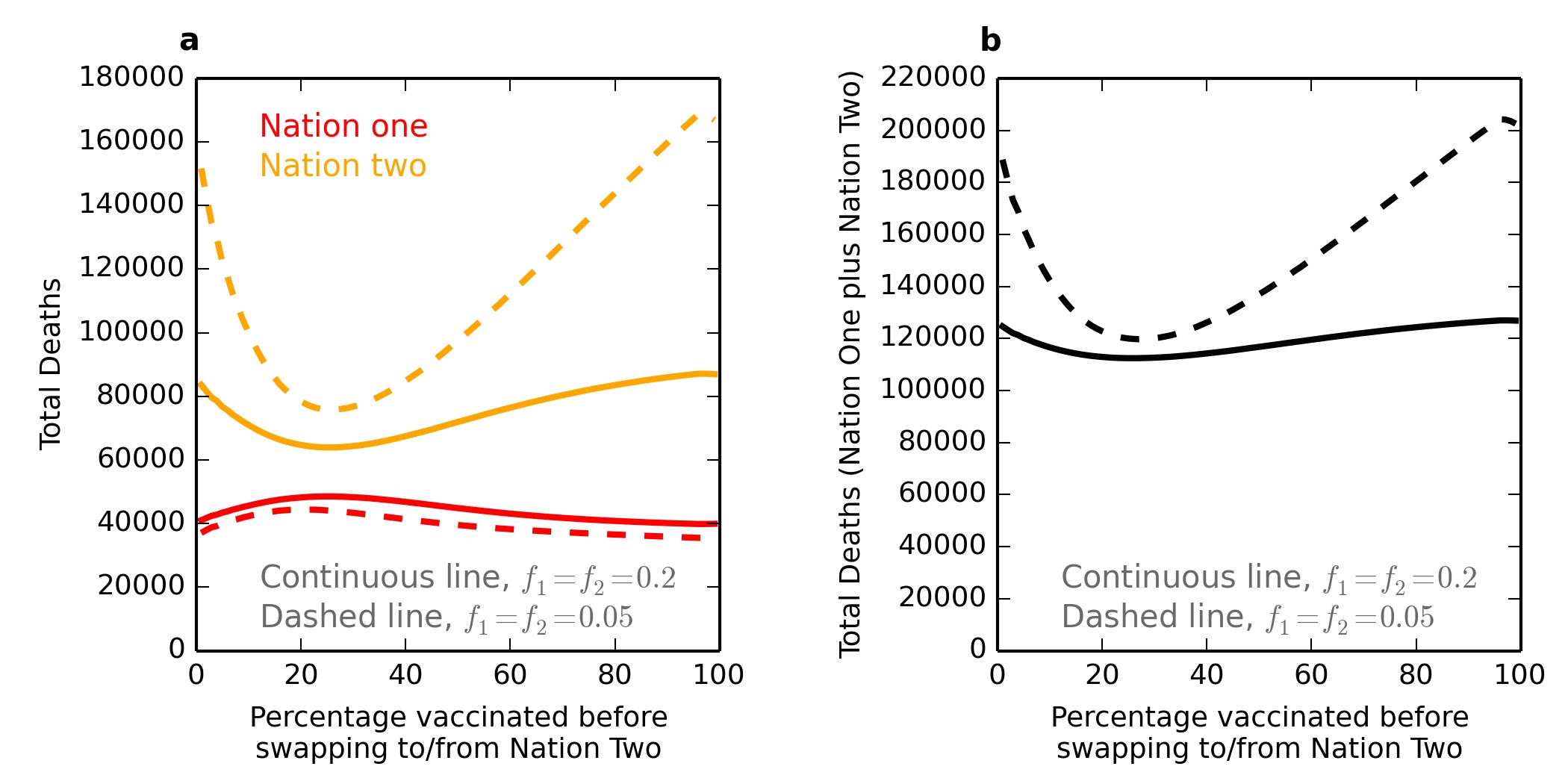

Supplement: Supplementary file 1 [file Data_Sheet_1.zip › supplemental_data_zip/python_code/figureS4/figureS4.jpg]

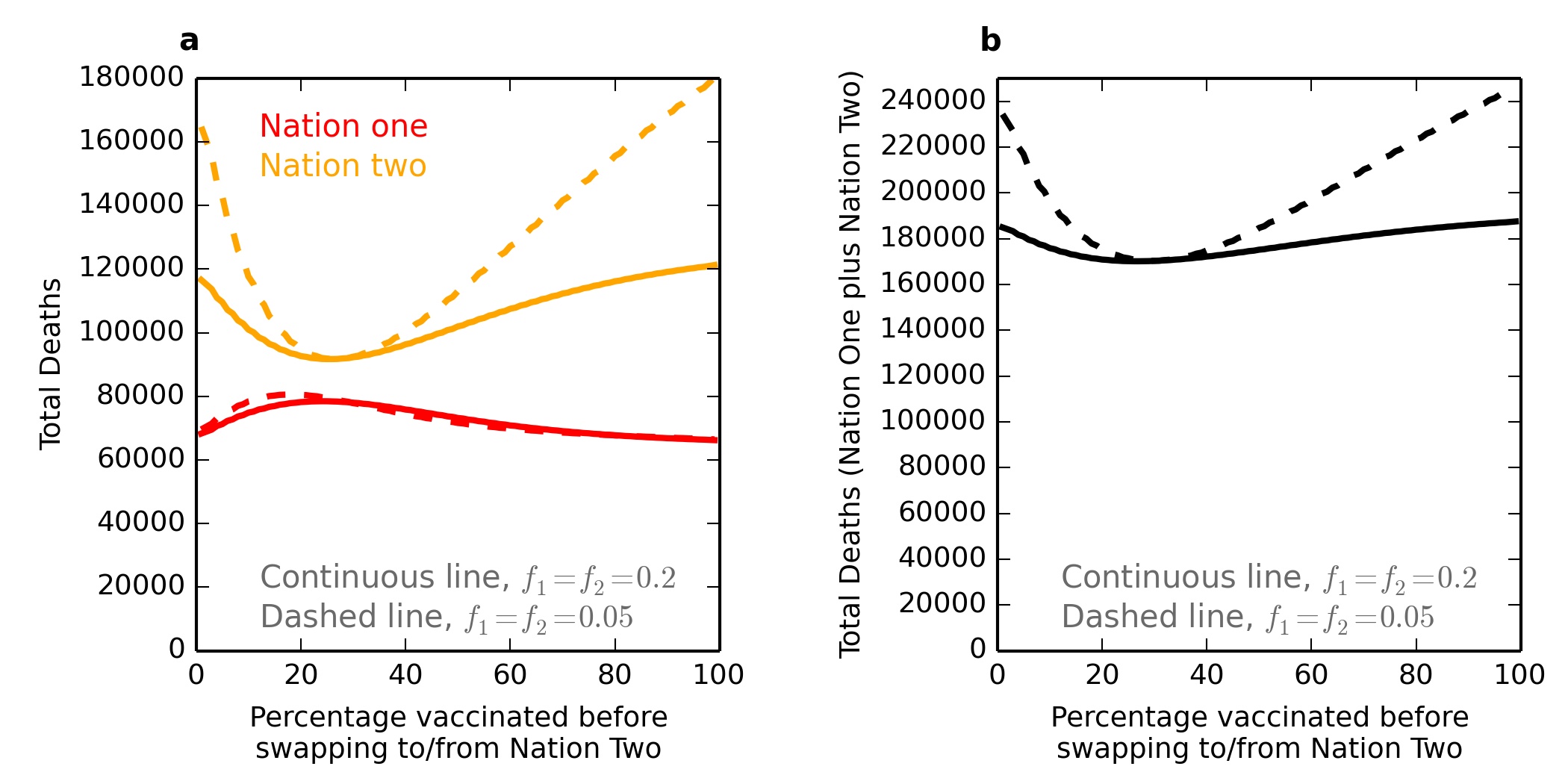

Supplement: Supplementary file 1 [file Data_Sheet_1.zip › supplemental_data_zip/python_code/figureS5/figureS5.jpg]

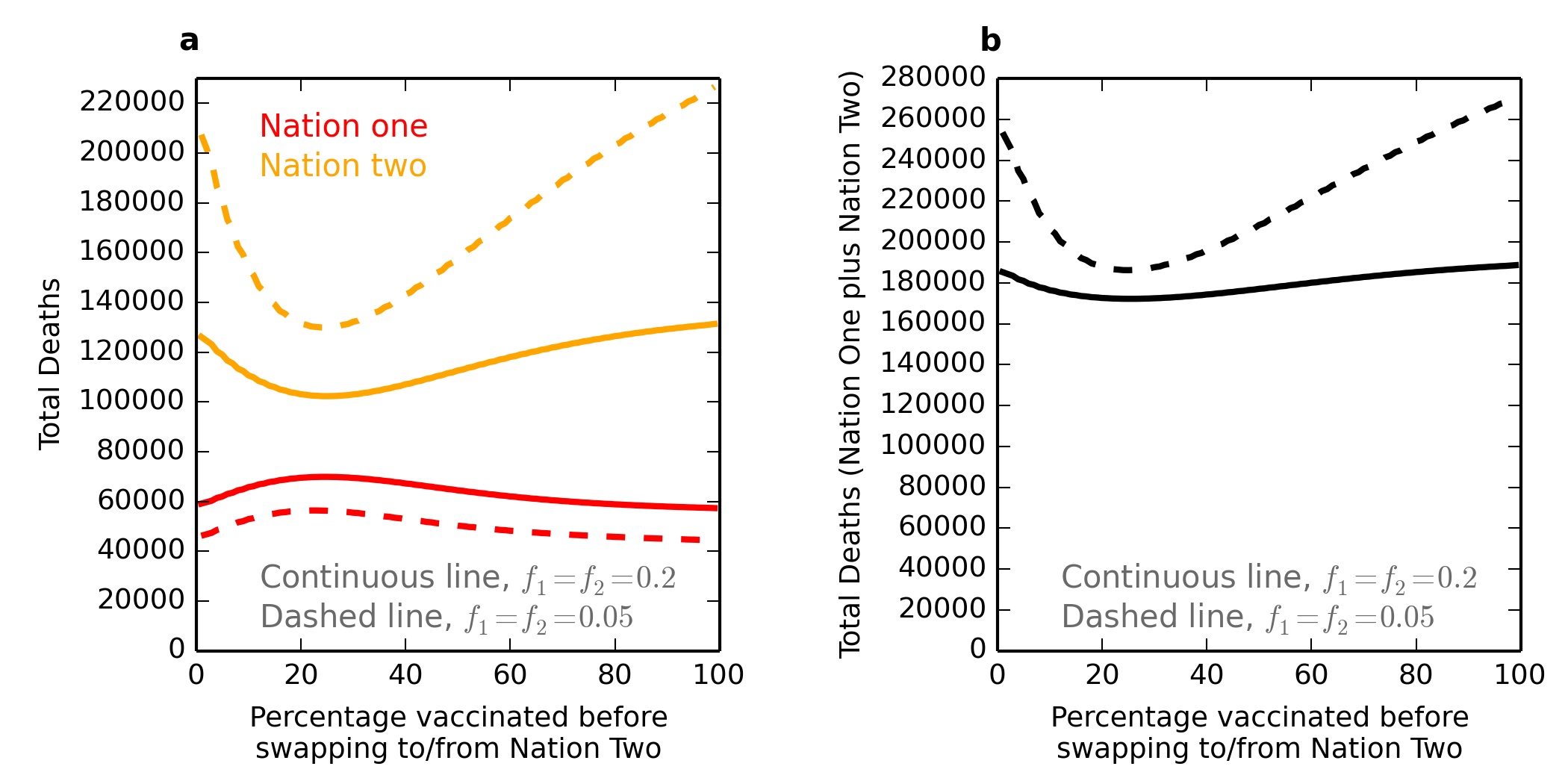

Supplement: Supplementary file 1 [file Data_Sheet_1.zip › supplemental_data_zip/python_code/figureS6/figureS6.jpg]
